# ONECUT2 is a driver of neuroendocrine prostate cancer

**DOI:** 10.1038/s41467-018-08133-6

**Published:** 2019-01-17

**Authors:** Haiyang Guo, Xinpei Ci, Musaddeque Ahmed, Junjie Tony Hua, Fraser Soares, Dong Lin, Loredana Puca, Aram Vosoughi, Hui Xue, Estelle Li, Peiran Su, Sujun Chen, Tran Nguyen, Yi Liang, Yuzhe Zhang, Xin Xu, Jing Xu, Anjali V. Sheahan, Wail Ba-Alawi, Si Zhang, Osman Mahamud, Ravi N. Vellanki, Martin Gleave, Robert G. Bristow, Benjamin Haibe-Kains, John T. Poirier, Charles M. Rudin, Ming-Sound Tsao, Bradly G. Wouters, Ladan Fazli, Felix Y. Feng, Leigh Ellis, Theo van der Kwast, Alejandro Berlin, Marianne Koritzinsky, Paul C. Boutros, Amina Zoubeidi, Himisha Beltran, Yuzhuo Wang, Housheng Hansen He

**Affiliations:** 1 Princess Margaret Cancer Centre, University Health Network, Toronto, M5G 1L7 ON Canada; 2 The Vancouver Prostate Centre, Vancouver General Hospital and Department of Urologic Sciences, The University of British Columbia, Vancouver, V6H 3Z6 BC Canada; 3 Department of Experimental Therapeutics, BC Cancer Research Centre, Vancouver, V5Z 1L3 BC Canada; 4 Department of Medical Biophysics, University of Toronto, Toronto, M5G 2M9 ON Canada; 5 Weill Cornell Medicine, New York, NY 10065 USA; 6 College of Life Sciences, Central China Normal University, Wuhan, 430079 Hubei People’s Republic of China; 7 College of Basic Medical Sciences, Dali University, Dali, 671000 Yunnan People’s Republic of China; 8 Department of Oncologic Pathology, Dana-Farber Cancer Institute, Boston, MA 02215 USA; 9 Department of Computer Science, University of Toronto, Toronto, M5T 3A1 ON Canada; 10 Ontario Institute for Cancer Research, Toronto, M5G 0A3 ON Canada; 11 Vector Institute, Toronto, M5G 1M1 ON Canada; 12 Memorial Sloan Kettering Cancer Center, New York, NY 10065 USA; 13 Department of Pathology, University of Toronto, Toronto, M5G 1L7 ON Canada; 14 Department of Radiation Oncology and Institute of Medical Science, University of Toronto, Toronto, M5T 1P5 ON Canada; 15 Department of Radiation Oncology, University of California at San Francisco, San Francisco, CA 94115 USA; 16 Department of Urology, University of California at San Francisco, San Francisco, CA 94115 USA; 17 Department of Medicine, University of California at San Francisco, San Francisco, CA 94115 USA; 18 Helen Diller Family Comprehensive Cancer Center, University of California at San Francisco, San Francisco, CA 94115 USA; 19 The Broad Institute, Cambridge, MA 02142 USA; 20 Department of Pharmacology & Toxicology, University of Toronto, Toronto, M5S 1A8 ON Canada

## Abstract

Neuroendocrine prostate cancer (NEPC), a lethal form of the disease, is characterized by loss of androgen receptor (AR) signaling during neuroendocrine transdifferentiation, which results in resistance to AR-targeted therapy. Clinically, genomically and epigenetically, NEPC resembles other types of poorly differentiated neuroendocrine tumors (NETs). Through pan-NET analyses, we identified ONECUT2 as a candidate master transcriptional regulator of poorly differentiated NETs. ONECUT2 ectopic expression in prostate adenocarcinoma synergizes with hypoxia to suppress androgen signaling and induce neuroendocrine plasticity. ONEUCT2 drives tumor aggressiveness in NEPC, partially through regulating hypoxia signaling and tumor hypoxia. Specifically, ONECUT2 activates SMAD3, which regulates hypoxia signaling through modulating HIF1α chromatin-binding, leading NEPC to exhibit higher degrees of hypoxia compared to prostate adenocarcinomas. Treatment with hypoxia-activated prodrug TH-302 potently reduces NEPC tumor growth. Collectively, these results highlight the synergy between ONECUT2 and hypoxia in driving NEPC, and emphasize the potential of hypoxia-directed therapy for NEPC patients.

## Abstract (web summary)

Neuroendocrine prostate cancer (NEPC) is characterized by loss of androgen receptor (AR) signaling during neuroendocrine transdifferentiation, resulting in resistance to AR-targeted therapy. Here they report ONECUT2 to drive NEPC tumorigenesis via regulation of hypoxia signaling and tumor hypoxia, and find hypoxia directed therapy to be effective in NEPC.

## Introduction

Neuroendocrine prostate cancer (NEPC) is a highly aggressive form of prostate cancer (PCa). Although it rarely arises de novo, NEPC may emerge from prostate adenocarcinoma (adeno-PCa) due to lineage plasticity induced by androgen receptor (AR)-targeted therapy^1,2^. A hallmark of NEPC is the loss of androgen receptor (AR) signaling during neuroendocrine transdifferentiation, resulting in resistance to AR-targeted therapy^3^. With the introduction of highly potent AR-targeted agents into the clinic, such as enzalutamide, the incidence of treatment-emergent NEPC (t-NEPC) is expected to escalate^4,5^. It has been reported recently that nearly one-fifth of metastatic CRPC develop small-cell neuroendocrine pathologic features after potent AR pathway inhibitor treatment^6^. Patients with NEPC have very limited therapeutic options, and the median overall survival is <1 year from time of diagnosis^7,8^. Understanding the molecular mechanisms driving t-NEPC is critical for improving therapeutic interventions for PCa patients.

NEPC resembles other poorly differentiated neuroendocrine tumors (NETs) such as small-cell lung cancer (SCLC), in both genomic and epigenetic alterations^1,9,10^. NETs, characterized by the presence of secretory granules and the production of hormones and amines^11,12^, are divided into well- and poorly differentiated subgroups based on their differentiation and proliferative index^1^. Well-differentiated NETs, which tend to be slow growing, show a pattern of trabecular, nested, or gyriform tumor cells with abundant neurosecretory granules^13,14^. In contrast, poorly differentiated NETs, which are more proliferative and aggressive, have diffuse or sheet-like architecture, abnormal nuclei and less cytoplasmic granularity^13,14^.

There are limited numbers of therapeutic options for patients with poorly differentiated NETs. Poorly differentiated NETs such as SCLC are typically treated with chemotherapy^15^. Most available systemic treatments are efficacious for only a limited time prior to the emergence of resistance. There is thus a great need for novel agents to improve remission rates and prolong overall survival. Understanding the common oncogenic mechanisms of poorly differentiated NETs could help identify common vulnerabilities across these aggressive tumors. Although a few pan-NET biomarkers have been identified, including chromogranin A and synaptophysin^11^, the master regulators that are critical in driving poorly differentiated NET have not been systematically assessed. Since a single transcription factor (TF) can modulate multiple downstream pathogenesis-related genes, identification of master TFs of NETs could pinpoint more efficacious therapeutic targets for NETs.

To fill this knowledge gap, we performed a pan-cancer analysis of poorly differentiated NET and non-NETs, and identified transcription factor ONECUT2 as a potential master regulator of poorly differentiated NETs. ONECUT2 is significantly upregulated in NEPC compared to adeno-PCa. Ectopic expression of ONECUT2 drives NE plasticity and induces hypoxia response genes in PCa cells. Importantly, NEPC tend to be more hypoxic than adeno-PCa, suggesting that hypoxia-directed therapy may benefit patients with NEPC.

## Results

### ONECUT2 is a potential master transcriptional regulator of poorly differentiated neuroendocrine tumors

**Fig. 1:**
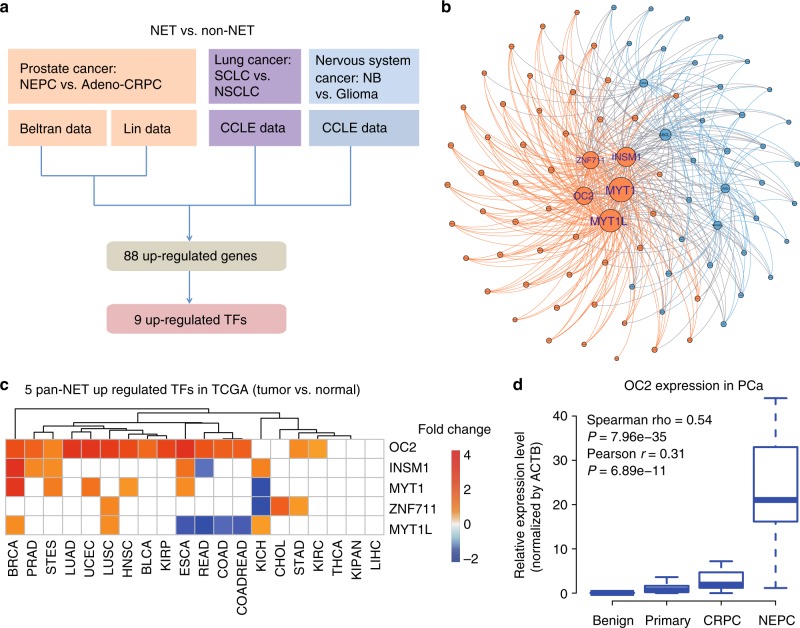
Pan-cancer analysis identifies ONECUT2 as a potential master transcriptional regulator of neuroendocrine tumors. **a** Schematic illustration of pan-NET analysis (see Methods section and Supplementary Figure 1a for details). Ninety-three genes, including nine transcription factors, were commonly upregulated in NETs compared to the non-NET counterparts within the same cancer types. NET neuroendocrine tumor, NEPC neuroendocrine prostate cancer, Adeno-CRPC castration-resistant prostatic adenocarcinoma, SCLC small-cell lung cancer, NSCLC non-small-cell lung cancer (exclusive of large cell lung cancer), NB neuroblastoma, CCLE Cancer Cell Line Encyclopedia. **b** Network analysis of nine TFs and 79 non-TF genes commonly upregulated in poorly differentiated NETs. These 88 genes were classified into two communities using GEPHI, as labeled by color (orange and cyan). **c** Dysregulation of the five TFs from the major community from panel **b** in 20 cancer types from TCGA. log_2_ transformed fold changes represent difference in expression between tumor and normal tissue. Colored cells indicate significant expression changes (absolute-value of the log_2_(fold change) > 1 and *P* < 0.05). The Wilcoxon rank sum test was used to calculate *P*-values. **d** ONECUT2 expression in benign prostate tissues and prostate tumors. RNA-Seq data of 52 benign prostate tissues and 333 primary PCa were retrieved from TCGA. RNA-Seq data of 34 adeno-CRPC and 15 NEPC were retrieved from the Beltran dataset. Expression levels as measured by RPKM were normalized by housekeeping gene ACTB

To identify potential master transcriptional regulators in NEPC, we performed differential mRNA abundance analysis in two independent castration-resistant prostate adenocarcinoma (adeno-CRPC) and NEPC transcriptome data sets^10,16^ (Fig. 1a). We identified 434 genes that are commonly upregulated in NEPC relative to adeno-CRPC (Supplementary Figure 1a). Considering the molecular similarity between NEPC and other types of poorly differentiated NETs^1,9,10^, we hypothesized that there are common master transcriptional regulators of poorly differentiated NETs. To test this hypothesis, we expanded our mRNA abundance analyses to two other cancer types, lung (i.e., SCLC and lung adenocarcinoma) and nervous system tumors (i.e., poorly differentiated neuroblastoma and glioma) using RNA-Seq data from Cancer Cell Line Encyclopedia (CCLE). With the same criteria, we identified 927 and 906 genes that are upregulated in the comparisons of poorly differentiated NETs vs. non-NETs in lung and nervous system cancer, respectively (Supplementary Figure 1a). Altogether, we identified a total of 88 genes that are upregulated in NETs vs. non-NETs in all three different cancer types, including 9 TFs (Supplementary Figures 1a-b and Supplementary Table 1). Importantly, well-characterized NET biomarkers like *CHGA, SYP* and *SYT4*^11^, were identified in our 88-gene list, confirming the accuracy of our analysis pipeline. Three of the 9 TFs (i.e., ASCL1, INSM1, and PROX1) have been reported to be either important regulators or biomarkers of NETs^17–19^, while three others (i.e., SIX2, MYT1 and MYT1L) are functional in neuronal development or endocrine tissues^20–22^.

To explore the potential regulatory relationships between the 9 TFs and the 79 non-TF genes, we performed a modulatory analysis using a co-expression network based on mRNA abundance correlation in the three NE tumor types (see Methods). The top community comprised 60% of genes, including five TFs (MYT1, MYT1L, ZNF711, ONECUT2, and INSM1) with top weighted degrees (Fig. 1b). These five TFs comprised the majority of nodes in this network, suggesting that they are more likely to be crucial NET transcriptional regulators.

To evaluate the potential role of these five TFs in tumorigenesis, we analyzed their mRNA expression in tumor and normal samples from 20 cancer types in TCGA dataset. ONECUT2 was the most commonly upregulated TF, showing increased mRNA levels relative to normal in 15 cancer types (Fig. 1c). *ONECUT2* is a member of the onecut family of transcription factors, known to be an important regulator of early retinal cell fate during development^23^. *ONECUT2* modulates the expression of oncogenic lncRNA *PCAT1* in prostate cancer, indicating its potential role in prostate cancer development^24^. However, its function in poorly differentiated NETs and NEPC is unknown.

### ONECUT2 expression increases during PCa progression

*ONECUT2* expression is significantly higher in human tumor samples of SCLC compared with lung adenocarcinoma (Supplementary Figure 2a), and in poorly differentiated neuroblastoma compared with glioma (Supplementary Figure 2b), consistent with the observation in CCLE cell line data. *ONECUT2* expression is also elevated in a NEPC patient-derived xenograft (PDX) model LTL331R, which was developed from a hormone-naive prostate adenocarcinoma PDX model LTL331 through castration (Supplementary Figure 2c).

Besides the human NETs and non-NETs, we also analyzed *Onecut2* expression in genetically engineered mouse models of PCa. PCa tumors initiated in *Pten* and *Rb1* double knockout (DKO) model showed increased lineage plasticity and features of NEPC compared with *Pten* knockout and single copy *Rb1* deletion (SKO) or normal prostate tissues (WT)^25^. *Onecut2* expression was significantly elevated in DKO compared with SKO and WT (Supplementary Figure 2d), consistent with the observation of *ONECUT2* upregulation in human NETs.

To further confirm its clinical relevance, we assessed *ONECUT2* expression from benign prostate tissues, primary adenocarcinoma and metastatic CRPC tumors in two independent clinical cohorts and a pooled cohort of TCGA and Beltran^10^. We determined that *ONECUT2* expression is significantly increased in CRPC compared to primary adenocarcinoma (Supplementary Figure 3a), while the largest difference is observed between NEPC and adeno-CRPC (Fig. 1d). Additionally, high level of *ONECUT2* in primary adenocarcinoma is a strong predictor of poor outcome as measured by biochemical recurrence-free survival in two clinical cohorts (Supplementary Figure 3b). Collectively, these data suggest that *ONECUT2* is associated with the progression of PCa.

### ONECUT2 regulates hypoxia signaling in NE-like PC3 cells

**Fig. 2:**
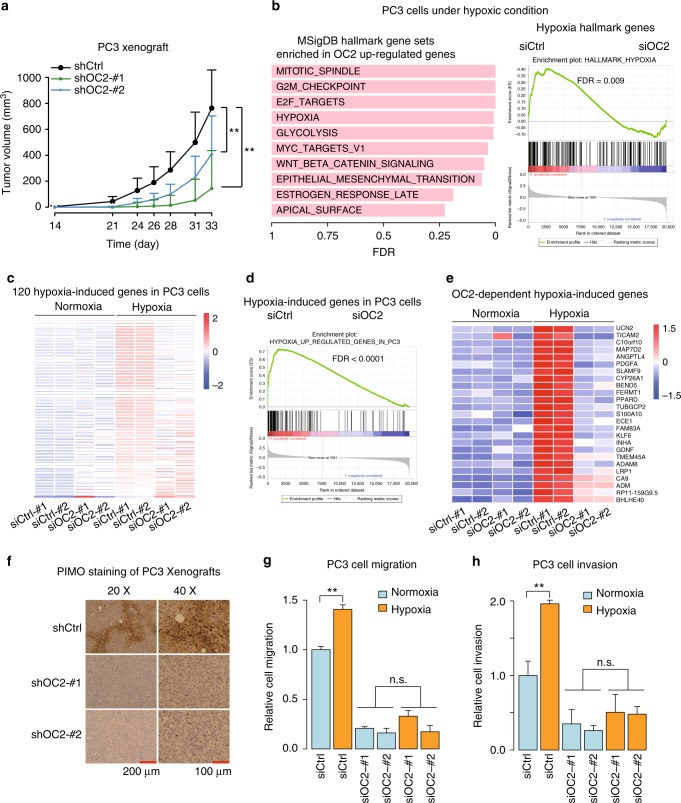
ONECUT2 regulates hypoxia signaling in NE-like PC3 cells. **a** Lentiviral knockdown of ONECUT2 suppresses tumor growth in PC3 xenograft models. *n* = 9 for shCtrl; *n* = 10 for shOC2-#1; *n* = 10 for shOC2-#2. **b** Left panel: top 10 MSigDB Hallmark Gene Sets enriched in ONECUT2 upregulated genes under hypoxic conditions. Right panel: GSEA enrichment plot for “Hypoxia” gene set. Genes were ranked by fold changes between control and ONECUT2 knockdown samples in descending order. **c** Heatmap shows relative expression of 120 hypoxia-induced genes (fold change > 2 and *P*-value < 0.05) in PC3 cells with and without ONECUT2 knockdown under normoxic and hypoxic conditions. **d** GSEA enrichment plot of hypoxia-induced genes in PC3 cells. Genes were ranked by fold changes between control and ONECUT2 knockdown samples in descending order. **e** Heatmap shows abundance of ONECUT2-dependent hypoxia-induced genes. **f** Representative images of Pimonidazole (PIMO) IHC staining of PC3 xenograft tumors with and without silencing of ONECUT2. **g**, **h** The capacity of PC3 cell migration and invasion with and without silencing of ONECUT2, under normoxic and hypoxic conditions in PC3 cells. *P*-values were calculated by mixed-effects models of repeated-measures ANOVA for **a**, Wilcoxon rank sum test for **c** and one-way ANOVA for **g** and **h**. n.s. not significant; *: *P* < 0.05; **: *P* < 0.01. Source data are provided as a Source Data file

To determine the function of ONECUT2 in NEPC, we silenced it in PC3 cells, an AR-negative PCa cell line with characteristics of prostatic small cell NE carcinoma^26^. ONECUT2 knockdown using two different siRNAs significantly decreased proliferation of NE-like PC3 cells (Supplementary Figure 4a-c). Consistently, silencing ONECUT2 using two different shRNAs significantly reduced subcutaneous tumor growth in PC3 xenografts (Fig. 2a; Supplementary Figure 4d). Similar effects on cell proliferation and tumor growth were observed in an additional NEPC cell line NCI-H660 and its cell line-derived xenograft models (Supplementary Figure 4e-f).

To further delineate the mechanisms underlying the function of ONECUT2 in NEPC, we performed RNA-Seq in PC3 cells with and without siRNA silencing of ONECUT2. We identified 67 and 93 genes significantly upregulated and down-regulated by ONECUT2, respectively (Supplementary Figure 5a). Gene Set Enrichment Analysis (GSEA) revealed that ONECUT2 targets genes were enriched with cell cycle related gene sets such as G2M_CHECKPOINT and E2F_TARGETS (Supplementary Figure 5b), in concordance with the function of ONECUT2 in regulating PC3 cell proliferation and tumor growth (Fig. 2a and Supplementary Figure 4c). Interestingly, we found enrichment of ANGIOGENESIS and HYPOXIA pathways in ONECUT2 regulated genes (Supplementary Figure 5b-c). Since hypoxia is strongly correlated with PCa progression and induction of NE plasticity in PCa^27,28^, we hypothesized that the function of ONECUT2 in NEPC is closely related with the regulation of cellular response to hypoxia.

To test this hypothesis, we analyzed ONECUT2 target genes in PC3 cells under hypoxic conditions using the ONECUT2 siRNA and RNA-Seq analysis as described above. GSEA identified very similar cell cycle related gene sets as with cells under normoxic conditions, while two hypoxia-related gene sets, HYPOXIA and GLYCOLYSIS, were markedly enriched in ONECUT2 upregulated genes under hypoxic conditions (Fig. 2b; Supplementary Figure 5d-e).

To identify hypoxia-responsive genes in PC3 cells, we profiled their transcriptomes under normoxic and hypoxic conditions using RNA-Seq. We identified 120 genes significantly induced by hypoxia (Fig. 2c). Interestingly, while only a small number of genes, such as CXCR4 and ARRDC3, tend to be modestly downregulated by ONECUT2 under both normoxic and hypoxic conditions, most of the 120 genes upregulated by ONECUT2 are specific under hypoxic conditions (Fig. 2c, d). Amongst the 120 genes, 25 were significantly upregulated by ONECUT2 under hypoxic conditions, including classical HIF-driven genes like *ADM* and *ANGPTL4* (Fig. 2e). These results suggest that ONECUT2 regulates hypoxia-induced gene expression in NEPC cells.

To determine the effect of ONECUT2 on tumor hypoxia, we analyzed hypoxia levels using pimonidazole (PIMO) staining in PC3 and NCI-H660 xenografts with and without silencing of ONECUT2. While tumors with control shRNAs were highly hypoxic, the hypoxia levels in ONECUT2 knockdown tumors were dramatically reduced (Fig. 2f; Supplementary Figure 6a-b). Silencing of ONECUT2 also significantly decreased PC3 cell proliferation under hypoxic conditions (Supplementary Figure 6c-d). Interestingly, while hypoxia treatment promoted PC3 cell migration and invasion, this effect was abrogated by ONECUT2 knockdown (Fig. 2g, h; Supplementary Figure 6e). These data suggest that ONECUT2 regulates tumor hypoxia and hypoxia-induced aggressive tumor biology in NEPC cells.

### ONECUT2 regulates HIF1α binding to the chromatin

**Fig. 3:**
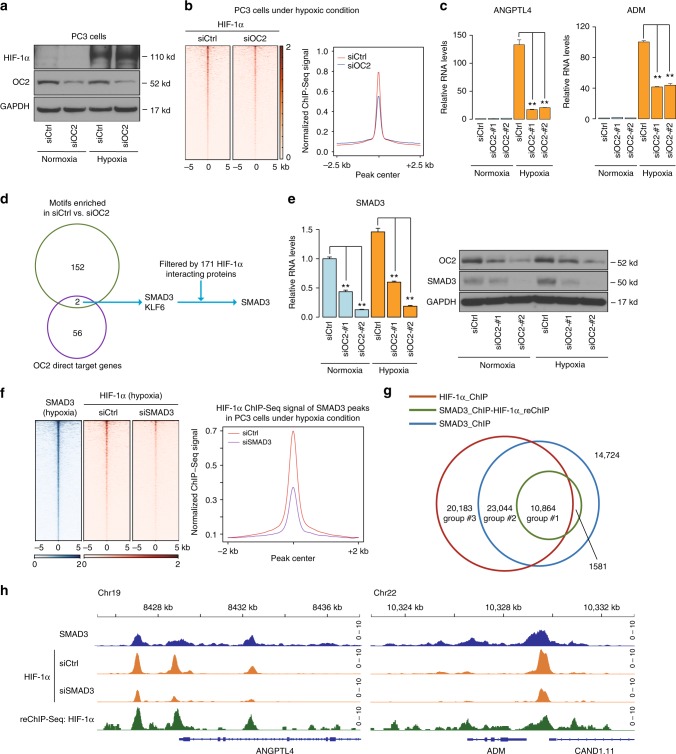
ONECUT2 modulates HIF1α binding to chromatin in NE-like PC3 cells. **a** Western blot of HIF1α and ONECUT2 in PC3 cells under normoxic and hypoxic conditions. Two different siRNAs targeting ONECUT2 were mixed together for knockdown experiments. **b** Left panel: heatmaps show HIF1α ChIP-Seq signal in PC3 cells under hypoxic conditions with and without knockdown of ONECUT2; right panel: pileup of HIF1α ChIP-Seq signals centered at HIF1α ChIP-Seq peaks center. **c** Expression of ANGPTL4 and ADM, two hypoxia-regulated genes, with and without knockdown of ONECUT2 in PC3 cells. **d** Schematic illustration of the analysis identifying SMAD3 as an ONECUT2 regulated HIF1α co-factor. Genes identified in motifs enriched in HIF1α binding sites and ONECUT2 target genes were further filtered by HIF1α interacting protein list from BioGRID. ONECUT2 target genes were defined as differentially expressed in ONECUT2 knockdown and control samples and with ONECUT2 binding sites nearby in PC3 cells under hypoxic conditions. **e** SMAD3 expression in response to ONECUT2 silencing in PC3 cells. **f** SMAD3 and HIF1α binding sites with and without silencing of SMAD3. **g** The overlap of SMAD3 ChIP-Seq, HIF1α ChIP-Seq and SMAD3-HIF1α ChIP-re-ChIP-Seq peaks. **h** SMAD3 ChIP-Seq, HIF1α ChIP-Seq and SMAD3-HIF1α ChIP-re-ChIP-Seq signal near the promoter regions of hypoxia-induced genes ANGPTL4 and ADM. Error bars indicate s.d. from at least two technical replicates. *P*-value is calculated by one-way ANOVA. **: *P* < 0.01. Source data are provided as a Source Data file

Since HIF1α plays a central role in transcriptional response to hypoxia^29,30^ and ONECUT2 regulates hypoxia signaling and tumor hypoxia, we next sought to characterize the interplay between HIF1α and ONECUT2 at transcriptional regulation. ONECUT2 knockdown did not affect HIF1α protein levels under hypoxic conditions, and vice versa (Fig. 3a; Supplementary Figure 7a). However, HIF1α genomic occupancy was globally decreased upon ONECUT2 knockdown as determined by ChIP-seq analyses (Fig. 3b), which was exemplified by binding sites near *ANGPTL4* and *ADM* (Supplementary Figure 7b). In addition, HIF1α binding sites were strongly enriched near (within 20 kb of transcription start sites (TSSs)) ONECUT2 upregulated genes (Supplementary Figure 7c). Consistent with this, RT-qPCR analysis demonstrated that the induction of *ANGPTL4* and *ADM* expression by hypoxia was significantly reduced by ONECUT2 knockdown (Fig. 3c). These data collectively suggest that ONECUT2 regulates hypoxia signaling through the regulation of HIF1α binding to the chromatin.

### ONECUT2 mediates HIF1α genomic binding through activation of SMAD3

We then explored the potential mechanism of how ONECUT2 regulates HIF1α chromatin binding in hypoxia response. Since ONECUT2 did not modulate HIF1α protein level, we compared ONECUT2 and HIF1α genomic binding sites (cistromes) in PC3 cell under hypoxic conditions. Only 8.4% of ONECUT2 and 7.6% of HIF1α binding sites overlapped with one another (Supplementary Figure 7d), which cannot explain the genome-wide reduction of HIF1α binding after ONECUT2 knockdown (Fig. 3b). Consistently, ONECUT2 or HIF1α immunoprecipitation followed by HIF1α or ONECUT2 western blot analyses did not detect interaction between ONECUT2 and HIF1α. We thus further tested whether ONECUT2 would activate HIF1α co-factors that modulate HIF1α binding. We first identified 58 ONECUT2 direct upregulated genes that fulfill two criteria: (1) significantly upregulated by ONECUT2 in PC3 cells under hypoxic conditions; (2) have ONECUT2 bindings within 20 kb from their TSSs. This gene list was then filtered by motifs enriched in HIF1α binding sites in control PC3 cells compared with ONECUT2 knockdown cells (Supplementary Table 2). Further filtering with HIF1α interacting proteins pinpointed SMAD3 as a candidate HIF1α co-factor activated by ONECUT2 (Fig. 3d).

We identified a ONECUT2 binding site near the SMAD3 promoter under both normoxic and hypoxic conditions (Supplementary Figure 8a), and consistent with RNA-Seq data, silencing of ONECUT2 dramatically reduced SMAD3 RNA and protein abundance in PC3 cells as determined by RT-qPCR and western blot analyses (Fig. 3e). After confirming that SMAD3 was regulated by ONECUT2, we tested whether SMAD3 was required for HIF1α chromatin binding. In PC3 cells under hypoxic conditions, SMAD3 knockdown dramatically reduced HIF1α ChIP-Seq signal globally (Fig. 3f, Supplementary Figure 8b), and nearly 70% of the SMAD3 ChIP-Seq peaks overlapped with that of HIF1α (Fig. 3g).

To further pinpoint the core regulatory regions of SMAD3 and HIF1α, we performed SMAD3 and HIF1α ChIP-re-ChIP experiments followed by high-throughput sequencing (Supplementary Figure 9a). This analysis identified 12,445 high-confidence binding sites, of which 87% overlapped with both HIF1α and SMAD3 ChIP-Seq peaks (Fig. 3g). In contrast, SMAD3 and HIF2α ChIP-re-ChIP-Seq identified only 324 peaks (2.7%) overlapped with both HIF2α and SMAD3 ChIP-Seq peaks (Supplementary Figure 9b), suggesting that SMAD3 directly interacts with HIF1α but not HIF2α. Among HIF1α ChIP-Seq peaks, the SMAD3-HIF1α core peaks (Fig. 3g, Group #1), as exemplified by the *ANGPTL4* and *ADM* locus (Fig. 3h, Supplementary Figure 9c), demonstrated strongest association with hypoxia-induced genes (Supplementary Figure 9d), further suggesting that ONECUT2-activated SMAD3 plays an important role in hypoxia signaling.

### ONECUT2 and hypoxia synergize to drive neuroendocrine plasticity

**Fig. 4:**
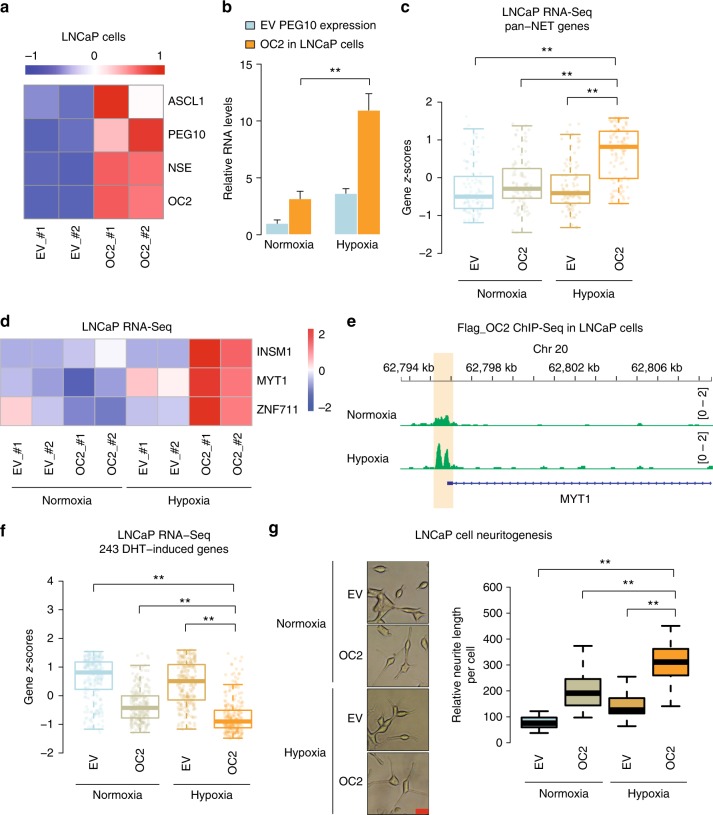
ONECUT2 synergizes with hypoxia in driving neuroendocrine plasticity in prostatic adenocarcinoma. **a** NEPC marker gene expression in LNCaP cells. mRNA abundance as measured by RT-qPCR assay was first normalized by housekeeping gene RPS28, and then *z*-score normalized; red represents higher signal and blue represents lower signal. **b** Expression of PEG10 in ONECUT2 overexpressing LNCaP cells under normoxic and hypoxic conditions. **c** Z-scores of the 92 pan-NET upregulated genes (ONECUT2 excluded) calculated in LNCaP RNA-Seq data. RNA-Seq experiments were performed in LNCaP cells with and without ONECUT2 overexpression under normoxic and hypoxic conditions. **d** Heatmap shows ONECUT2 overexpression and hypoxia synergistically promote expression of the core pan-NET transcriptional factors. **e** ONECUT2 ChIP-Seq signal at MYT1 promoter under normoxic and hypoxic conditions. **f** Z-scores of the 243 DHT-induced genes calculated in LNCaP RNA-Seq data. RNA-Seq experiments were performed in LNCaP cells with and without ONECUT2 overexpression under normoxic and hypoxic conditions. **g** Neuritogenesis analysis in LNCaP cell with and without overexpression of ONECUT2 under normoxic and hypoxic conditions. Error bars in **b** indicate s.d. from four technical replicates. *P*-values are calculated from a Student’s *t*-test in **a** and **b** and Wilcoxon rank sum test in **c**, **f**, and **g**. *: *P* < 0.05. **: *P* < 0.01. EV empty vector control, OC2 ONECUT2 overexpression. Source data are provided as a Source Data file

Having demonstrated the function of ONECUT2 in NEPC, we further explored its role in prostate adenocarcinoma. Transient overexpression of ONECUT2 in LNCaP cells, an adeno-PCa cell line, was sufficient to induce the expression of NE marker genes including *ASCL1*, *PEG10*, and *NSE* (Fig. 4a, Supplementary Figure 10a), indicating that ONECUT2 is a regulator of NE plasticity.

Recent studies have reported that hypoxia can induce NE plasticity in PCa^28,31,32^. Consistent with these reports, LNCaP cells under hypoxic conditions demonstrated significantly higher levels of NE marker genes compared with the cells under normoxic conditions (Supplementary Figure 10b). To investigate whether there are synergies between ONECUT2 and hypoxia in driving NE plasticity, we evaluated the expression of NE marker genes under normoxic and hypoxic conditions with or without overexpression of ONECUT2. Interestingly, ONECUT2 overexpression and hypoxia synergized to drive elevated PEG10 mRNA abundance (Fig. 4b). To further explore the synergy between ONECUT2 and hypoxia in the development of NEPC, we performed RNA-Seq in LNCaP cells with or without ONECUT2 overexpression under normoxic and hypoxic conditions. Consistent with the results in PC3 cells, ONECUT2 overexpression in LNCaP cells promotes the expression of hypoxia hallmark genes under hypoxic conditions (Supplementary figure 10c-d). In addition, hypoxia treatment demonstrated strong synergy with ONECUT2 in activating expression of the 92 pan-NET upregulated genes (Fig. 4c; excluding ONECUT2). It is worth noting that three (i.e., MYT1, INSM1, and ZNF711) out of the four core pan-NET TFs (Fig. 1b, excluding ONECUT2) identified by our pan-NET analysis were synergistically upregulated by ONECUT2 overexpression and hypoxia treatment (Fig. 4d). Consistent with the mRNA abundance data, ONECUT2 bindings at the promoter of *MYT1* and enhancers of *INSM1* and *ZNF711* were enhanced under hypoxic compared to normoxic conditions (Fig. 4e; Supplementary Figure 11a-b). In addition, ONECUT2 and hypoxia synergistically suppressed the expression of androgen-induced genes in LNCaP cells (Fig. 4f), consistent with the clinical observation of reduced AR signaling during NE transdifferentiation. Recent studies suggested that NE transdifferentiation from adeno-CRPC to NEPC is through an intermediary stem-like state, of which the cells exhibit epithelial–mesenchymal transition (EMT) and stem cell-like features^25,33–35^. ONECUT2 and hypoxia synergistically induced both EMT and stem cell-related gene signatures in LNCaP cells (Supplementary Figure 11c-d), suggesting high lineage plasticity of those cells.

We next tested whether ONECUT2 and hypoxia collaborated to drive NE-like cell morphology in adeno-PCa cells. ONECUT2 overexpression or hypoxia treatment alone was able to induce neuritogenesis in LNCaP cells, and the combination has a much stronger effect (Fig. 4g). This observation was validated in adeno-CRPC V16A cells (Supplementary Figure 12a-b). Knockdown of *HIF1α* completely abolished ONECUT2 and hypoxia-induced NE marker genes expression (Supplementary Figure 12b), which indicated the essential role of HIF1α in ONECUT2 and hypoxia-induced NE plasticity.

TP53 and RB1 are two most frequently mutated genes in NEPC, and TP53/RB1 double deletion has been reported to promote lineage plasticity of adeno-PCa^25,33^. We investigated whether ONECUT2 synergizes with TP53/RB1 double knockdown to further promote NE plasticity. In V16A cells, either ONECUT2 overexpression or TP53/RB1 double knockdown alone induced neuritogenesis and NE marker genes expression, but no strong additive effect was observed in combination of ONECUT2 overexpression and TP53/RB1 double knockdown (Supplementary Figure 12c-e). TP53/RB1 double knockdown or RB1 deletion resulted in increased expression of ONECUT2 in V16A and genetically engineered mouse models, respectively (Supplementary Figure 2d and 12e), suggesting the NE plasticity driven by TP53/RB1 deletion may partially be mediated through activation of ONECUT2.

### NEPC with ectopic ONECUT2 expression is highly hypoxic

**Fig. 5:**
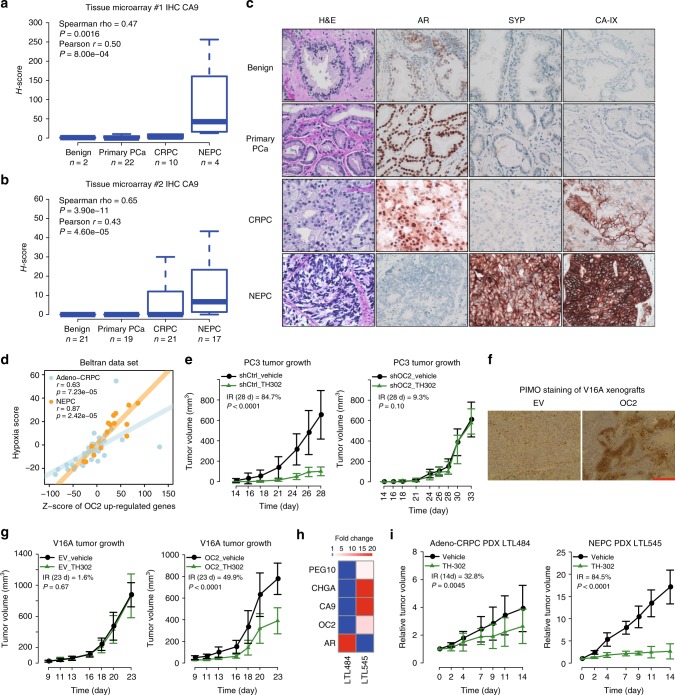
NEPC is highly hypoxic and sensitive to hypoxia-directed treatment. **a**, **b** Hypoxia marker gene CA9 IHC staining in two independent tissue microarray sets. The combined H-score, taking into account of both staining intensity and percentage, was used to quantify CA9 protein levels. (c) Representative images of tissue microarray analysis of AR, SYP, and CA9 IHC staining in benign prostate, primary PCa, adeno-CRPC, and NEPC tissues. Scale bar = 50 μm. **d** Correlation between ONECUT2 upregulated genes (summarized in *z*-scores) and hypoxia scores in Beltran adeno-CRPC/NEPC dataset. **e** PC3 xenograft tumor growth in response to hypoxia-activated prodrug TH-302 treatment, with and without silencing of ONECUT2. *n* = 7 for shCtrl_Vehicle; *n* = 6 for shCtrl_TH302; *n* = 8 for shOC2_Vehicle; *n* = 7 for shOC2_TH302. **f** Representative images of PIMO IHC staining of V16A xenograft tumors with and without overexpression of ONECUT2. Scale bar = 200 μm. **g** V16A xenograft tumor growth in response to TH-302 with and without overexpression of ONECUT2. *n* = 7 for EV_Vehicle; *n* = 8 for EV_TH302; *n* = 6 for OC2_Vehicle; *n* = 6 for OC2_TH302. **h** Relative expression levels of related genes in the adeno-CRPC (LTL484) and NEPC (LTL545) PDX models determined by microarray analysis. **i** TH-302 treatment suppresses tumor growth in NEPC patient-derived xenografts. *n* = 4 for vehicle group and *n* = 4 for TH-302 group in NEPC PDXs; *n* = 5 for vehicle group and *n* = 5 for TH-302 group in adeno-CRPC PDXs. In **e**, **g**, and **i**, inhibition rate (IR) of tumor growth was calculated as (Tumor volume ^Vehicle^−Tumor volume ^TH-302^)/Tumor volume ^Vehicle^. *P* values were determined by mixed-effects models of repeated-measures ANOVA for **e**, **g**, and **i**. Source data are provided as a Source Data file

ONECUT2 overexpression synergizes with hypoxia to drive NE plasticity and aggressive tumor biology in NEPC. This therefore raises the question of whether NEPC is an inherently highly hypoxic tumor subtype. To address this question, we analyzed the expression of hypoxia marker gene CA9 by immunohistochemistry (IHC) in two independent tissue microarrays (TMAs) with 38 and 78 prostate tumor and benign tissue samples, respectively. In both TMAs, CA9 staining was significantly stronger in NEPC compared to primary adeno-PCa and adeno-CRPC (Fig. 5a–c), indicating that NEPC is more hypoxic than prostate adenocarcinoma. We further calculated hypoxia scores in two clinical cohorts of transcriptome data using a 32-gene hypoxia signature^36^. In agreement with our tissue microarray analysis, NEPC samples exhibited significantly higher hypoxia scores compared to the adeno-CRPC samples (Supplementary Figure 13a). Consistently, in the RNA-Seq data of genetically engineered mouse model of PCa, DKO demonstrated the highest hypoxia score compared to SKO and WT (Supplementary Figure 13b).

To evaluate the relationship between ONECUT2 and prostate tumor hypoxia, we used the ONECUT2 up-regulated genes in PC3 cells under hypoxic condition as a signature of ONECUT2 activity. Only one gene was common between the hypoxia signature and ONECUT2 targets (Supplementary Figure 13c), excluding the potential bias caused by overlapped genes. ONECUT2 scores were positively correlated with hypoxia scores in primary prostate adenocarcinoma in two independent clinical cohorts, with *r* = 0.5 and 0.6 respectively (Supplementary Figure 13d). This correlation slightly increased in adeno-CRPC samples (*r* = 0.627) and was highest in NEPC samples (*r* = 0.871) (Fig. 5d). These results, together with previous PIMO staining data in PC3 xenografts (Fig. 2f), indicate that ONECUT2 activity is increasingly correlated with PCa tumor hypoxia in the progression to NEPC.

Finally, having demonstrated that NEPC is more hypoxic than adeno-CRPC, we hypothesized that NEPC is more sensitive to hypoxia-directed treatment than adeno-CRPC. We therefore tested the tumor inhibition efficacy of a hypoxia-activated prodrug, TH-302^37^, in V16A and PC3 xenografts. TH-302 treatment dramatically reduced PC3 xenograft tumor growth (tumor inhibition rate = 84.7%) but did not affect that of V16A xenograft (tumor inhibition rate = 1.6%; Fig. 5e, g, left panels). Importantly, silencing of ONECUT2 in PC3 xenografts almost completely abolished the tumor inhibition effect of TH302, and overexpression of ONECUT2 sensitized the V16A xenografts to TH302 (Fig. 5e, f, right panels). In line with the tumor inhibition effects, PIMO staining in PC3 xenografts was much stronger than in V16A xenografts (Figs. 2f and 5f**;** Supplementary Figure 6a and 13e), and ONECUT2 knockdown reduced tumor hypoxia in PC3 xenografts, while ONECUT2 overexpression increased tumor hypoxia in V16A xenografts (Figs. 2f and 5f).

We further assessed the effect of TH-302 on tumor growth of adeno-CRPC and NEPC patient-derived xenograft models. Weekly treatment of TH-302 dramatically inhibited NEPC PDX tumor growth in vivo compared to control group (tumor inhibition rate = 84.5%), without obvious weight loss (Fig. 5h, i; Supplementary Figure 13f), but only had a moderate inhibitory effect on adeno-CRPC tumor growth (tumor inhibition rate = 29.2%, Fig. 5i). Furthermore, PIMO staining confirmed that TH-302 treatment diminished tumor hypoxia in NEPC PDX tumors (Supplementary Figure 14). Altogether, these results suggest that hypoxia-directed therapy is a promising treatment option for patients with NEPC.

## Discussion

While the mechanisms by which NEPC arises from prostate adenocarcinoma are poorly understood, recent studies suggest that transdifferentiation occurs from prostatic adenocarcinoma and not normal prostate glands^10,38^. In addition, t-NEPC cases, which can be induced from adeno-CRPC, are emerging with the widespread usage of potent AR-targeted agents in clinical practice^5^. Therefore, alternative therapeutic options are becoming a pressing need for targeting the molecular mechanisms driving NEPC. To identify key drivers of NEPC, we conducted a pan-NET analysis by integrating four datasets comparing poorly differentiated NET vs. non-NET, including two of PCa. ONECUT2 was the top candidate regulator from our analysis.

In this study, we focused on PCa, and showed that ONECUT2 is upregulated in primary PCa compared with normal prostate tissues, and further upregulated in metastatic PCa. Similarly, higher ONECUT2 expression is also correlated with worse clinical outcomes in PCa patients. These observations indicate that ONECUT2 may play important roles in a broad spectrum of PCa progression. By using in vitro cell line models, we reveal that ONECUT2 regulates the aggressive tumor biology in NEPC, at least partially through activating SMAD3-HIF1α signaling. Finally, using cell line and patient-derived xenograft models, we provide evidence that a hypoxia-directed treatment potently inhibits NEPC tumor growth, and thus may serve as a promising treatment option for patients with NEPC.

We demonstrated that ONECUT2 synergizes with hypoxia to induce lineage plasticity towards a NE-like phenotype as measured by both morphological changes and NE marker genes expression in adeno-PCa cells. However, we acknowledge that our findings are limited by in vitro cell line models. Further validation in genetically engineered mouse or in pre-clinical PCa organoid models is warranted. The elevated ONECUT2 expression in adeno-CRPC tumors compared with primary PCa tumors also raises a possibility that some adeno-CRPC tumors may possess NE plasticity and molecular features and are at high risk for either progression or transition to NEPC. Blocking ONECUT2 and/or targeting hypoxia may prevent the development of NEPC for these patients at high risk and thus is worth exploring in future studies.

While we only validated the function of ONECUT2 in PCa, its function may not be limited to NEPC. Indeed, our analysis showed that ONECUT2 expression was elevated specifically, in poorly differentiated lung cancers and nervous system NETs compared with non-NETs. These data indicate that ONECUT2 may also have important function in poorly differentiated NETs of other cancer types besides NEPC. Therefore, the function of ONECUT2 in other types of NETs merits further investigation, especially in SCLC and poorly differentiated neuroblastoma. Additionally, ONECUT2 shows significant upregulation in cancers derived from multiple human tissues, indicating that ONECUT2 may play a critical role in tumorigenesis pan-cancer wide.

Besides ONECUT2, our analysis also identified eight other TFs upregulated in pan-NETs, including 4 TFs correlated with expression of the majority of other NET upregulated genes. Some of these TFs, such as ASCL1 and INSM1, have been well characterized in NET biology and survival^17,39^. However, for the remaining TFs, their functions have only been partially revealed in neuronal development or endocrine tissues, which are potentially related with NE differentiation/plasticity in tumors. Further studies of the function of these TFs may identify new drivers and therapeutic targets for poorly differentiated NETs.

Most solid tumors contain regions of hypoxia. The level of hypoxia varies in different tumor types and is a strong negative prognostic and predictive factor in multiple types of cancer, with particularly strong evidence in prostate cancer^40,41^. Selective tumor hypoxia targeting by hypoxia-activated pro-drug TH-302 in combination with immunotherapy is being evaluated in clinical studies in multiple cancer types, including locally advanced or metastatic PCa (ClinicalTrials.gov Identifier: NCT00743379). Our results showed that NEPC is more hypoxic than adeno-CRPC, and hypoxia targeting by TH-302 dramatically reduced tumor growth of NEPC cell line and patient tumor derived xenografts. Furthermore, ONECUT2 is also expressed in certain normal tissues, such as brain, liver and pancreas, based on GTEx RNA-Seq data. Therefore, direct targeting of ONECUT2 may lead to unfavorable side effects, while targeting of ONECUT2-dependent tumor hypoxia could be a more convenient and optimal choice for NEPC patients. Altogether, our study demonstrated the importance of ONECUT2-hypoxia axis in the development of NEPC, and revealed hypoxia-directed treatment as a novel therapeutic option for NEPC patients.

## Methods

### Pan-cancer analysis

For pan-NET analysis, two PCa data sets generated by Beltran et al. and Lin et al. were used to identify genes up-regulated in NEPC compared with adeno-CRPC^10,16^. For the Lin dataset, one new NEPC PDX microarray data (LTL545) was added to increase statistical power. SCLC cell lines from CCLE (https://portals.broadinstitute.org/ccle/home) were used as NE type and NSCLC cell lines as non-NE type lung cancers. Neuroblastoma cell lines from CCLE were used as NE type and glioma as non-NE type nervous system tumors. RNA-Seq data of corresponding cell lines were retrieved from CCLE to identify genes up-regulated in NE-type compared with non-NE type cancers. Wilcoxon test was used to calculate p-value in every comparison and Benjamini-Hochberg adjustment was conducted to assess the false discovery rates (FDR) of multiple comparisons. Genes co-up-regulated (fold change >2 and FDR < 0.05) in NE vs. non-NE comparisons of all the four data sets were subjected to the following network analysis. For the network analysis, Pearson correlations were calculated between the co-up-regulated 9 TFs and 84 non-TF genes using their expression data in CCLE SCLC, CCEL neuroblastoma and Beltran NEPC data sets. Pearson correlations between the 10 TFs were also added to the network. Correlation count was defined by *r* > 0.5 in one dataset. Correlation count 1, 2, or 3 indicates two genes are correlated (*r* > 0.5) in one, two or three data sets, respectively. Co-expression network was constructed based on the correlations and visualized by Gephi 0.9.1. Edge weights were defined by correlation counts. Node sizes were defined by weighted degrees. Communities in the network were detected by the modularity function^42^ in Gephi. Three NET vs. Non-NET data sets were used for further validation of ONECUT2 expression: (a) 29 SCLC RNA-Seq data from Rudin et al. dataset^43^ and 535 TCGA-LUAD RNA-Seq data from Genomic Data Commons Data Portal (https://portal.gdc.cancer.gov/); (b) 157 TARGET-NBL RNA-Seq data from Genomic Data Commons Data Portal and 529 TCGA-LGG RNA-Seq data from Genomic Data Commons Data Portal; (c) gene expression data during the transformation from prostatic adenocarcinoma (LTL331) to NEPC (LTL331R) from GSE59984^35^. For pan-cancer tumor vs. normal analysis, TCGA level 3 RNA-Seq data were downloaded from Firehose Broad GDAC (https://gdac.broadinstitute.org/, 2016-01-28 batch). For differential expression analysis, one cancer type was used only when the number of normal samples was more than 5.

### Cell culture and treatment

LNCaP, NCI-H660, and PC3 cell lines were obtained from the American Type Culture Collection (ATCC). V16A was established by Dr. Amina Zoubeidi’s laboratory. All prostate cancer cell lines were cultured as recommended by ATCC. No mycoplasma contamination was detected in these cell lines using MycoAlert™ Mycoplasma Detection Kit (LT07-118, Lonza). PC3 cell proliferation was determined by AlamarBlue™ (DAL1025, ThermoFisher) staining. PC3 cell migration and invasion were determined by transwell migration and matrigel invasion assays as described previously^44^. Briefly, PC3 cells were first transfected with ONECUT2 siRNAs and incubated for 48 h. Then, 1 × 10^4^–1 × 10^5^ cells were transferred to 24-well transwell chambers (BD Biosciences, USA) followed by 24 h normoxia or hypoxia treatment. Cells that penetrated membrane were stained with crystal violet. For hypoxia treatment, cells were incubated in hypoxia chamber at 0.2% O_2_. Fiji plugin Simple Neurite Track was used to quantify the neurite length of V16A and LNCaP cells following the developer’s instruction^45^.

### siRNA transfection

siRNAs targeting *ONECUT2, HIF1A, SMAD3*, and control siRNAs were purchased from ThermoFisher. Lipofectamine RNAiMAX transfection reagent (13778150, ThermoFisher) was used for siRNA transfection following the manufacturer’s instructions. The siRNA target sequences were listed in Supplementary Table 3.

### Vectors and lentiviral transfection

ONECUT2 overexpression vector pCMV6-XL5-hONECUT2 was kindly provided by Dr. Merlin Crossley. To construct lentiviral vector of pLKO.1-TRC-shONECUT2, two shRNA oligonucleotides targeting different regions of ONECUT2 were inserted to pLKO.1-TRC vector (10878, Addgene), respectively. Sequences of ONECUT2 shRNAs are listed in Supplementary Table 3. To construct lentiviral vector of pLenti-CMV-Puro-DEST-Flag-ONECUT2, ONECUT2 protein coding DNA sequence was inserted to entry vector pENTR4-FLAG (17423, Addgene) first and then transferred to pLenti-CMV-Puro-DEST (17452, Addgene). pLenti-C-Myc-DDK-P2A-Puro-ONECUT2 (RC211951L3) and pLenti-C-Myc-DDK-P2A-Puro (PS100092) were purchased from OriGene. Lentiviral particle production and infection were performed as described previously^46^. In brief, lentiviral vectors were co-transfected with psPAX2 and pMD2G vectors into HEK293T cells. Supernatants were collected at 24 and 48 h after transfection and stored in −80 °C. For infection, 5 × 10^4^ cells per well were seeded in six-well plates and infected with lentiviral supernatant on the following day.

### Chromatin immunoprecipitation (ChIP) and ChIP-Seq

ChIP assay was performed using LNCaP and PC3 cells. Protein A (88845, ThermoFisher) and G (88847, ThermoFisher) Dynabeads were mixed at a 1:1 ratio, and pre-incubated with antibodies for 3 hrs before immunoprecipitation. LNCaP and PC3 cells were cross-linked by 1% formaldehyde for 10 min and then quenched with 125 mM glycine. After a cold PBS wash, the nuclear fraction was extracted in 10 mL of LB1 buffer (50 mM HEPES–KOH, pH 7.5; 140 mM NaCl; 1 mM EDTA; 10% Glycerol; 0.5% IGEPAL CA-630; 0.25% Triton X-100) for 10 min at 4 ℃. Nuclear fraction was then pelleted and resuspended in 10 mL of LB2 buffer (10 mM Tris–HCL, pH 8.0; 200 mM NaCl; 1 mM EDTA; 0.5 mM EGTA) at 4 ℃ for 5 minutes. Nuclear fraction was pelleted again and resuspended in LB3 buffer (10 mM Tris–HCl, pH 8; 100 mM NaCl; 1 mM EDTA; 0.5 mM EGTA; 0.1% Na–Deoxycholate; 0.5% N-lauroylsarcosine; Protease inhibitor cocktail). Nuclear fraction was then sonicated in a water bath sonicator (Diagenode bioruptor) to generate chromatin fragments at ~300 bp. 1/10 volume of 10% Triton X-100 was added to chromatin lysate. Chromatin lysate was cleared by centrifugation and 1/10 of supernatant was taken as input DNA. The rest chromatin lysate was divided equally to antibody-conjugated beads tubes and rotated at 4 ℃ overnight. Antibodies used for ChIP assays are anti-HIF1α (NB100-479, Novus), anti-HIF2α (NB100-122, Novus), anti-SMAD3 (ab40854, Abcam) and anti-Flag (F3165, Sigma). The beads were washed in RIPA buffer (50 mM Tris, pH 7.6, 150 mM NaCl, 1 mM EDTA, 0.1% SDS, 1% IGEPAL CA630, 0.5% sodium deoxycholate) and Elution buffer (0.1 M NaHCO3; 1% SDS; Proteinase K) was used to reverse cross-linking of DNA-protein complex at 65 ℃ for 8–16 h. DNA was purified by phenol–chloroform extraction and subjected to ChIP-Seq library preparation. For ChIP-Seq, 5 ng of DNA (ChIP-enriched or input) was used for library creation with the Rubicon ThruPLEX-FD kit and sequenced (75 bp single-end reads). All ChIP-Seq data were aligned using Bowtie2 (version 2.2.1) to the human genome of build version NCBI37/HG19. MACS2 was used for peak calling with the parameter “–SPMR” on^47^. Resultant bedgraph files were converted to big wiggle files by UCSC bedGraphToBigWig tool. The significantly enriched motifs in siCtrl HIF1α ChIP-Seq peaks relative to siOC2 HIF1α ChIP-Seq peaks were identified by CentriMo^48^.

### re-ChIP and re-ChIP-Seq

For re-ChIP assays, complexes from the primary immunoprecipitation were eluted from the beads by 37 °C incubation in 20 mM dithiothreitol for 30 min. The eluates were then diluted at least 30~50 fold with dilution buffer (2 mM EDTA, 150 mM NaCl,1% Triton X-100, 20 mM Tris-HCl, pH 8.1) and subjected to a second immunoprecipitation reaction. Tris-EDTA buffer with 1% SDS was used to elute re-ChIPed DNA. re-ChIP library preparation and sequencing were performed in the same way as ChIP-Seq. For re-ChIP-Seq analysis, after reads alignment, the primary ChIP-Seq bam file was used as the control for re-ChIP-Seq bam file in the peak calling by MACS2. To generate normalized bedgraph files of re-ChIP-Seq data, re-ChIP-Seq bedgraph file and primary ChIP-Seq bedgraph file were subjected to MASC2 bdgcmp sub-command with the option “–method = FE”. Peak overlapping as shown by Venn diagram was determined by R package ‘ChIPpeakAnno’^49^.

### Western blotting

Western blotting was performed as described previously^50^. The blots were first incubated with anti-ONECUT2 (21916-1-AP, Proteintech; ab28466, Abcam), anti-SMAD3 (ab40854, Abcam), anti-HIF1α (NB100-479, Novus), anti-H3 (ab1791, Abcam) or anti-GAPDH (ab1791, Abcam), and then with 1:10000 anti-rabbit secondary antibody (7074, Cell Signaling).

### Mouse xenograft of PC3 cells and Patient-derived xenografts

All animal experiments were approved by the University Health Network Animal Care Committee (ID: AUP4714). Murine xenograft transplants were established directly from V16A cells with ONECUT2 overexpression or PC3 cells with ONECUT2 depletion using lentiviral shRNAs as described above. Briefly, 1 × 10^6^ PC3 or V16A cells resuspended in 100 μL PBS/Matrigel (1:1) were subcutaneously injected into the upper right flank of male immunodeficient NOD-SCID mice between 5 and 8 weeks of age. Tumor growth was monitored by measuring two perpendicular diameters and calculating tumor volume (mm^3^) using the formula *d*^2^*D/2 where *d* and *D* are the shortest and longest diameter in mm, respectively.

NEPC Patient-Derived Xenografts (PDX) LTL545 and adeno-CRPC PDX LTL484 were established in Dr. Wang lab^16^. These PDXs were propagated and maintained by subcutaneous passages in NOD-SCID mice. During all PDX implantation procedures, animals were anesthetized and their temperature was maintained at 38 °C with a heating lamp. After PDX implantation, mice were randomized to vehicle or TH-302 treatment groups. After PDX tumor volumes reached ~50 mm^3^_,_ TH-302 was administered by intraperitoneal injection at 75 mg/kg once per week. Tumor growth monitor and volume calculation were performed as described above. To assess the effect of TH-302 treatment, PDX tumor volumes were normalized to the initial volumes at the beginning of TH-302 or vehicle treatment. To assess the treatment effects on tumor growth, tumor volume data at different time points were subjected to mixed-effects models for repeated-measures ANOVA by R package ‘nlme’.

### RNA-Seq

Total RNA of PC3 and LNCaP cells was extracted using RNeasy Mini Kit (74106, Qiagen) following manufacturer’s procedure. Whole transcriptome sequencing libraries were prepared using TruSeq® Stranded mRNA Library Prep Kit (RS-122-2101, Illumina, San Diego, CA, USA) by following the manufacturer’s instructions. RNA-Seq libraries were sequenced on a HiSeq2500 at Princess Margaret Genomic Centre. The trimmed reads were aligned to human genome hg19 with STAR (version 2.4.2a)^51^ and gene expression was then quantified using the reads per kilobase per million mapped reads (RPKM) method by Cufflinks (version 2.1.1) with GENCODE v24 GRCh37 GTF file. Gene Set Enrichment Analysis (GSEA)^52^ was used to evaluate the association of ONECUT2 expression with known pathways. GSEA was performed using the hallmark gene sets from version 4.0 of the molecular signature database (MSigDB) which are coherently expressed signatures derived by aggregating many MSigDB gene sets to represent well-defined biological states or processes.

### Hypoxia score and ONECUT2-upregulated gene *z*-score

Hypoxia score and ONECUT2-upregulated gene *z*-score of each participant were defined as the sum of *z*-scores of corresponding signature genes. Hypoxia score is based on a 32-gene prostate cancer-specific hypoxia signature^36^. ONECUT2 score is based on the 123 ONECUT2-upregulated genes in PC3 cells under hypoxic conditions. For each gene, *z-score* *=* (*x−μ*)/*σ*; *x* indicates pre-normalized gene expression level, *μ* indicates study mean of gene expression and *σ* indicates study standard deviation of gene expression.

### Immunohistochemistry

Preparation of paraffin-embedded tissue sections and immunohistochemical analyses of PC3 and V16A xenografts and NEPC PDXs were carried out at histology core facility of Princess Margaret Cancer Centre. Paraffin sections at 4 μm thickness were dried at 60 °C oven for 2 hours before staining. The immunohistochemistry (IHC) was performed according to the manufacture’s guidelines using BenchMark XT-an automated slide strainer (Ventana Medical System) with standard antigen retrieval (CC1, pH 8.0, #950-124). For PIMO staining, 60 mg/kg PIMO HCl (Hypoxyprobe™-1, Hypoxyprobe) was intraperitoneal injected into tumor bearing mice and tumors were collected 60 minutes later. Mouse monoclonal anti-PIMO antibody (MAb1, Hypoxyprobe, 1:400 dilution), rabbit polyclonal anti-CA9 antibody (NB100-417, Novus, 1:1000 dilution), rabbit polyclonal anti-SYP antibody (ab32127, Abcam, 1:1000 dilution) and rabbit polyclonal anti-CHGA antibody (ab15160, Abcam, 1:1000 dilution) were used for immunohistochemistry. Biotinylated anti-rabbit IgG (Vector, BA-1000) was added to slides at 1:200 for 12 minutes. The primary-secondary antibody complex was then visualized with Ventana iView DAB Detection Kit (#760-091). The slides were counterstained with Harris hematoxylin, dehydrated in graded alcohol, cleared in xylene and coverslipped in Permount.

### Tissue microarray

In this study, we conducted two independent tissue microarray analysis using TMAs from the Vancouver Prostate Cancer Tissue Bank (TMA #1) and the Weill Cornell Medicine (TMA #2).

TMA #1 consists a total of 38 prostate tumor and benign tissue samples, which were obtained from the Vancouver Prostate Centre Tissue Bank. H&E slides were first reviewed and desired areas were marked also on their correspondent paraffin blocks. A TMA was created by taking double 1 mm cores from matching selected areas of paraffin blocks using a semi-automated tissue microarrayer from Pathology Devices TMA arrayer with Leica M50 stereomicroscope. Immunohistochemical staining was conducted by Ventanaautostainer model Discover XT™ (Ventana Medical System, Tuscan, Arizona) with enzyme labeled biotin streptavidin system and solvent-resistant DAB Map kit by using 1/200 concentration of CA9 mouse monoclonal antibody. All stained slides were digitalized with the SL801 autoloader and Leica SCN400 scanning system (Leica Microsystems; Concord, Ontario, Canada) at magnification equivalent to×40. The images were subsequently stored in the SlidePath digital imaging hub (DIH; Leica Microsystems) of the Vancouver Prostate Centre. Prostate cancer specimens were obtained from patients following a protocol approved by the Clinical Research Ethics Board of the University of British Columbia and the BC Cancer Agency (all patients signed a consent form approved by the Ethics Board).

For TMA #2, benign prostate (*n* = 21), prostate adenocarcinoma (*n* = 19), castration-resistant prostate adenocarcinoma (CRPC) (*n* = 21) and neuroendocrine prostate cancer (NEPC) (*n* = 17) samples were evaluated and scored at WCM for expression of androgen receptor (AR), synaptophysin (SYP), and CA9. Antibodies used were: CA9 (Novus Biologicals #NB100-417, Citrate-based antigen retrieval, 1:1000) AR (Biogenex, #MU256-UC, EDTA-based antigen retrieval, 1:800), SYP (Leica PA0299, EDTA-based antigen retrieval). Prostate cancer specimens were obtained from patients following a protocol approved by the Weill Cornell Medicine (WCM) Institutional Review Board (IRB) with informed consent.

Each core of the TMA was scored as % of cells with 0 = no expression, 1 = low expression, 2 = moderate expression, and 3 = high expression of CA9. H-score analysis was based on % of positive cells and intensity of staining in positive cells (range 0–300). Images where taken at ×40, scales bar 50 μm.

## Supplementary information

Supplementary Information

Description of Additional Supplementary Files

Supplementary Data 1

## Data Availability

The RNA-seq and ChIP-seq raw sequence tags and processed bed files reported in this manuscript have been submitted to the National Centre for Biotechnology Gene Expression Omnibus (GEO) database (accession no. GSE106305). The data that support the findings of this study are available from the corresponding author upon reasonable request. A reporting summary for this Article is available as a Supplementary Information file. The source data underlying Figs. 2g, h, 3a, c, e, 4b, and 5e, g, h and Supplementary Figs 4a, 4b, 4c, 4f, 6c, 7a, 9c, 10a, 11b and 11e are provided in Supplementary Data 1.
