# Supplementary material for: ONECUT2 is a driver of neuroendocrine prostate cancer
Source: Nat Commun. 2019 Jan 17;10:278. doi: 10.1038/s41467-018-08133-6 (PMC6336817; doi:10.1038/s41467-018-08133-6)
Supplement: Supplementary file 1 — Supplementary Information [file 41467_2018_8133_MOESM1_ESM.pdf]

SUPPLEMENTARY INFORMATION

ONECUT2 is a Driver of Neuroendocrine Prostate Cancer

Guo *et al*

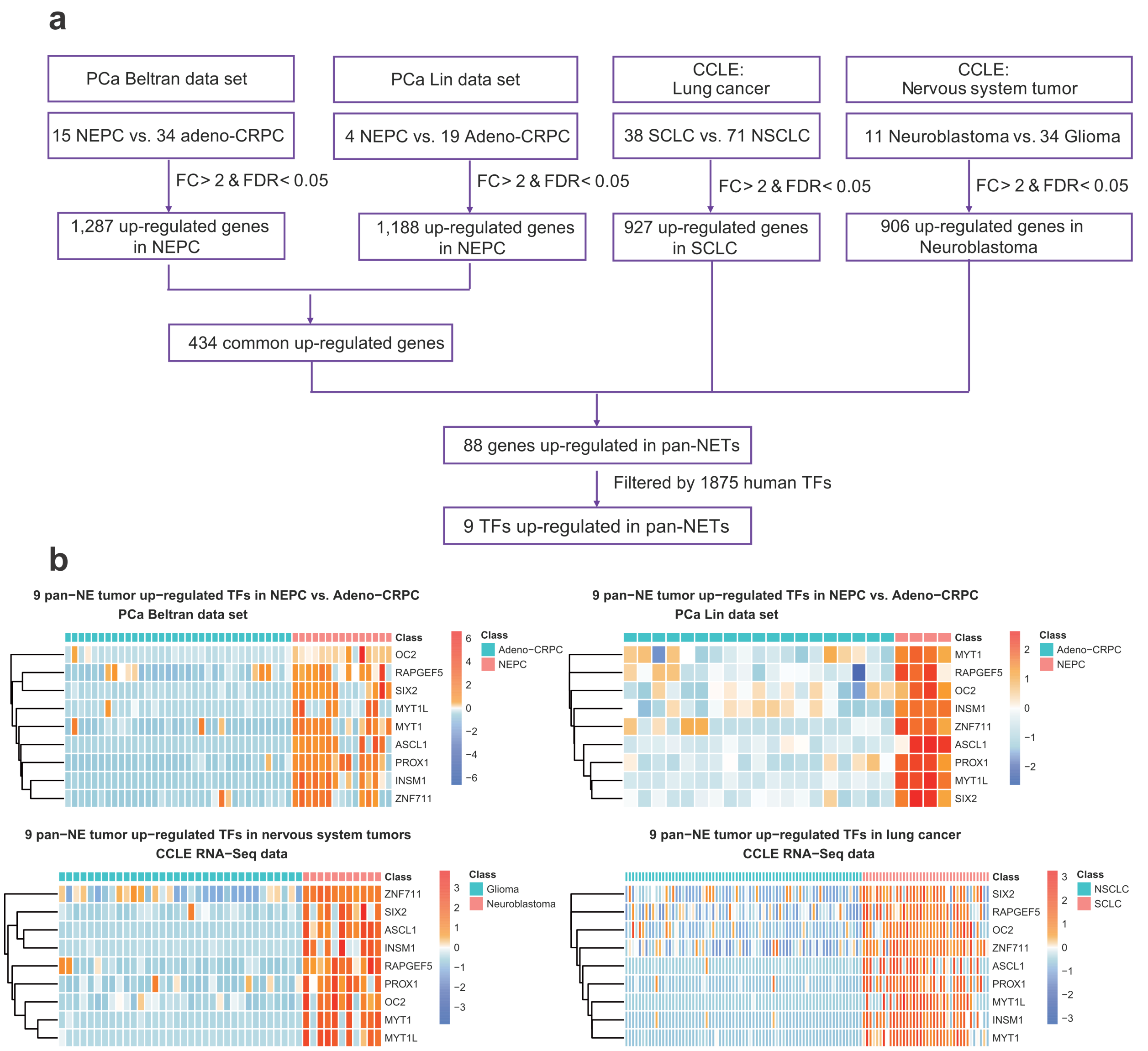

Supplementary Figure 1. Pan-cancer analysis identifies master transcriptional regulators of neuroendocrine tumors. (a) Schematic diagram of pan-NET analysis (see Method section for details). NET: neuroendocrine tumor; NEPC: neuroendocrine prostate cancer; Adeno-CRPC: castration-resistant prostate adenocarcinoma; SCLC: small cell lung cancer; NSCLC: non-small cell lung cancer (exclusive of large cell lung cancer). CCLE: Cancer Cell Line Encyclopedia; FC: fold change; FDR: false discovery rate. (b) Heatmap shows expression of the 9 pan-NET up-regulated TFs in the four data sets used in pan-NET analysis.

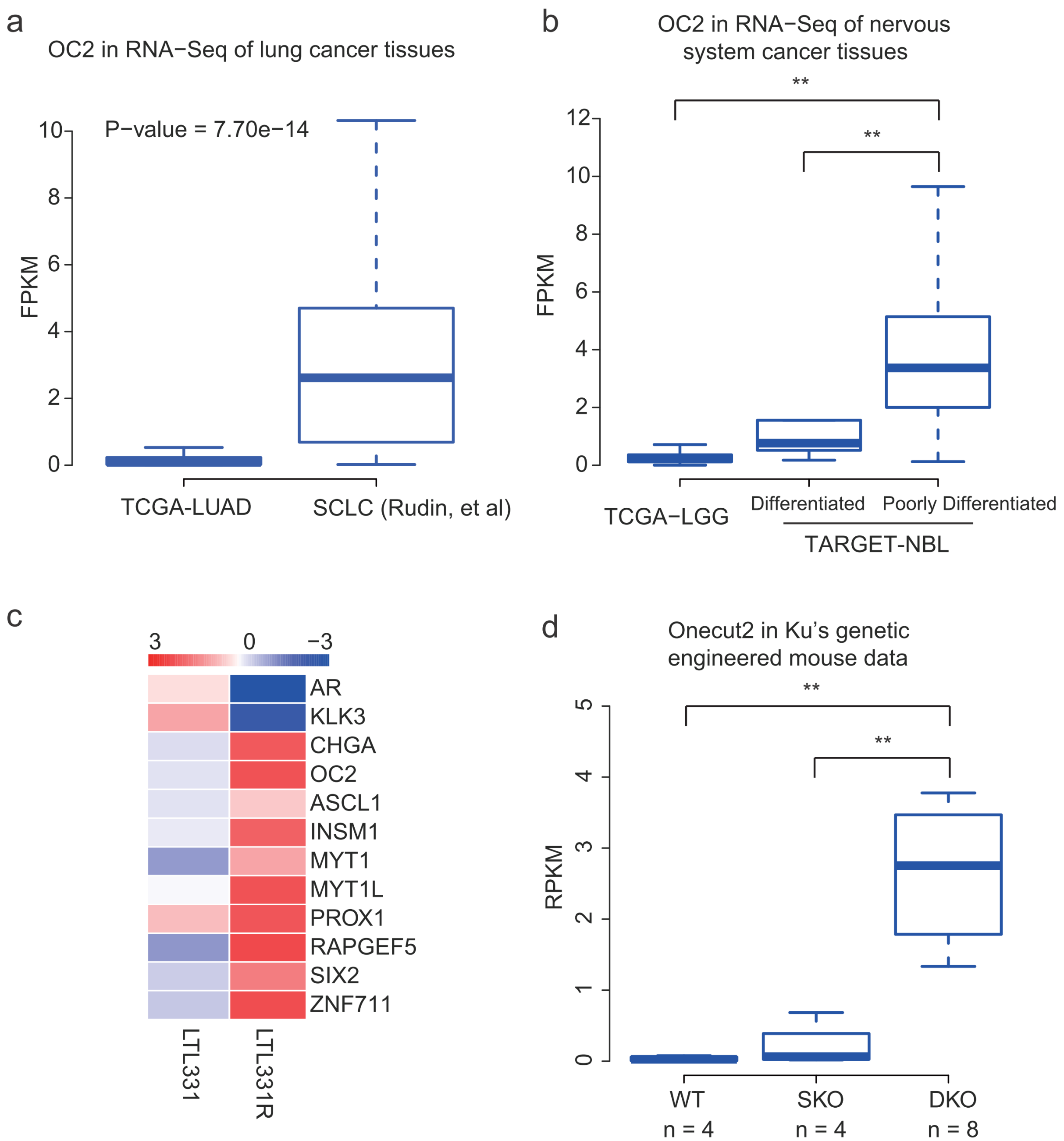

Supplementary Figure 2. ONECUT2 is up-regulated in NETs compared with non-NETs in multiple additional data sets. (a) ONECUT2 is up-regulated in SCLC (RNA-Seq data from Rudin data set) compared with LUAD (Lung adenocarcinoma RNA-Seq data from TCGA). (b) ONECUT2 is up-regulated in NBL (Neuroblastoma RNA-Seq data from TARGET) compared with LGG (Lower grade glioma RNA-Seq data from TCGA). NBL samples are grouped by their differentiation status. (c) ONECUT2 and NE marker genes are overexpressed in a NEPC patient-derived xenograft (PDX) model LTL331R, compared with the hormone-naive prostatic adenocarcinoma PDX LTL331. (d) Onecut2 is up-regulated in DKO (Pten and Rb1 double knockout; more NE-like than SKO) PCa tumors compared with SKO (Pten knockout, single

copy Rb1 deletion) PCa tumors or WT (normal prostate tissues). RNA-Seq data is from GSE90891. P-values were calculated from Wilcoxon rank sum test; **: P < 0.01.

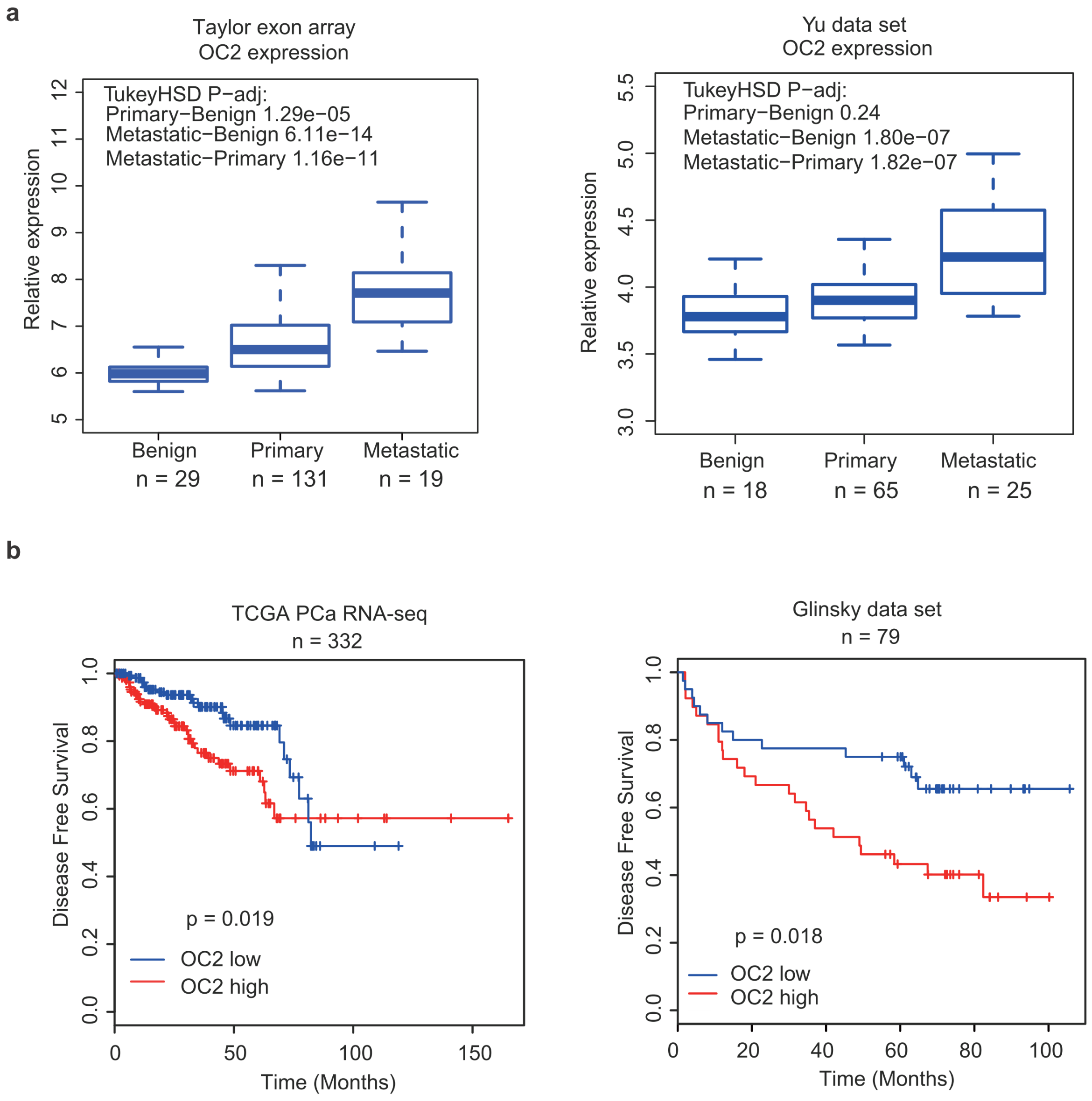

Supplementary Figure 3. ONECUT2 is correlated with PCa progression and patient outcome. (a) ONECUT2 mRNA is up-regulated in metastatic prostate cancer compared with primary prostate cancer in two independent PCa data sets. (b) High ONECUT2 expression is correlated with poor PCa patient outcome in two independent PCa data sets. P-values were determined by log-rank test.

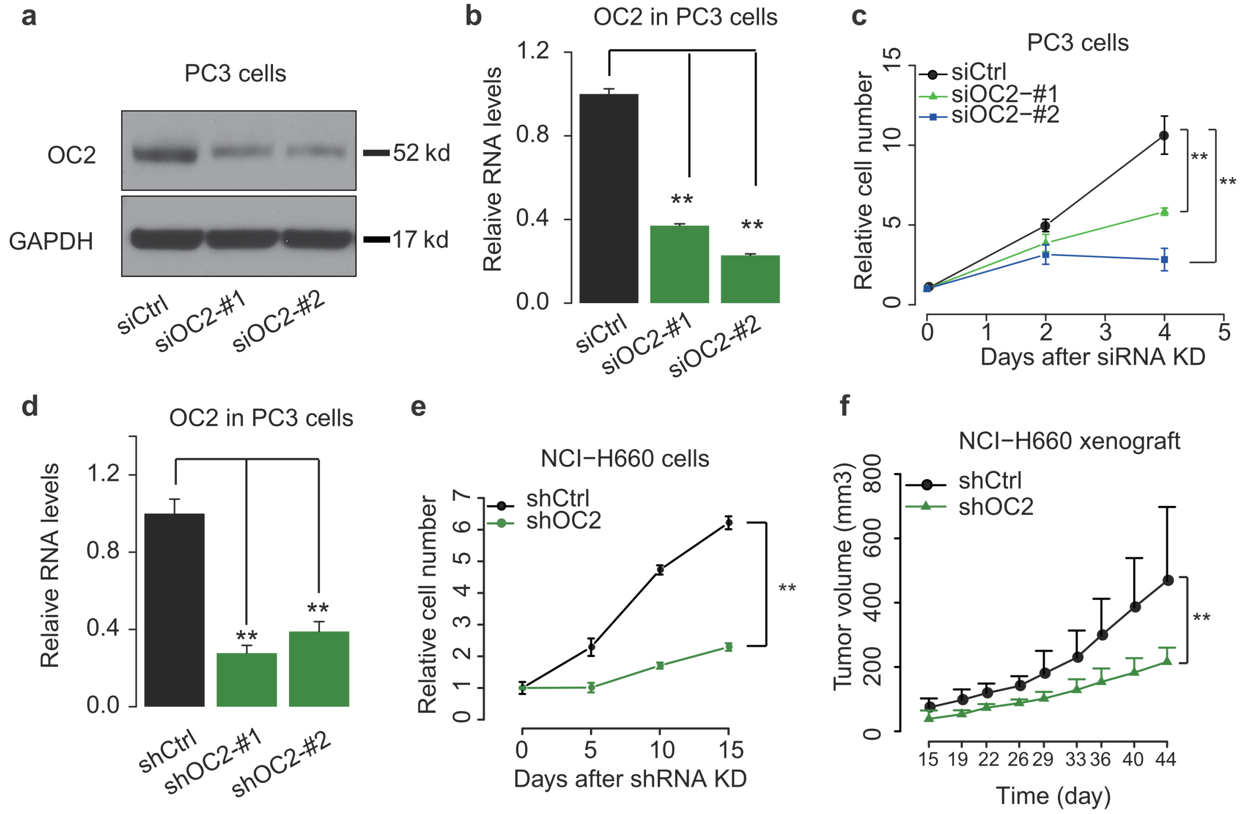

Supplementary Figure 4. ONECUT2 knockdown assays in PCa cell lines. (a-b) ONECUT2 western blotting (a) and RT-qPCR (b) in PC3 cells with and without silencing of ONECUT2. Two different siRNAs targeting ONECUT2 were used for knockdown assays. (c) PC3 cell proliferation was determined by alamar blue staining after ONECUT2 siRNA knockdown. Error bars indicate s.d. from four technical replicates. (d) ONECUT2 RT-qPCR in PC3 cells with and without silencing of ONECUT2. Two different lentiviral shRNAs targeting ONECUT2 were used for knockdown assays. (e) NCI-H660 cell proliferation was suppressed by ONECUT2 shRNA knockdown. (f) NCI-H660 xenograft tumor growth was suppressed by ONECUT2 shRNA knockdown. n = 8 for shCtrl group and n = 8 for shOC2 group. P-values were calculated from one-way ANOVA for (a-e) and mixed-effects models of repeated-measures ANOVA for (f); **: $P < 0.01$. Source data are provided as a Source Data file.

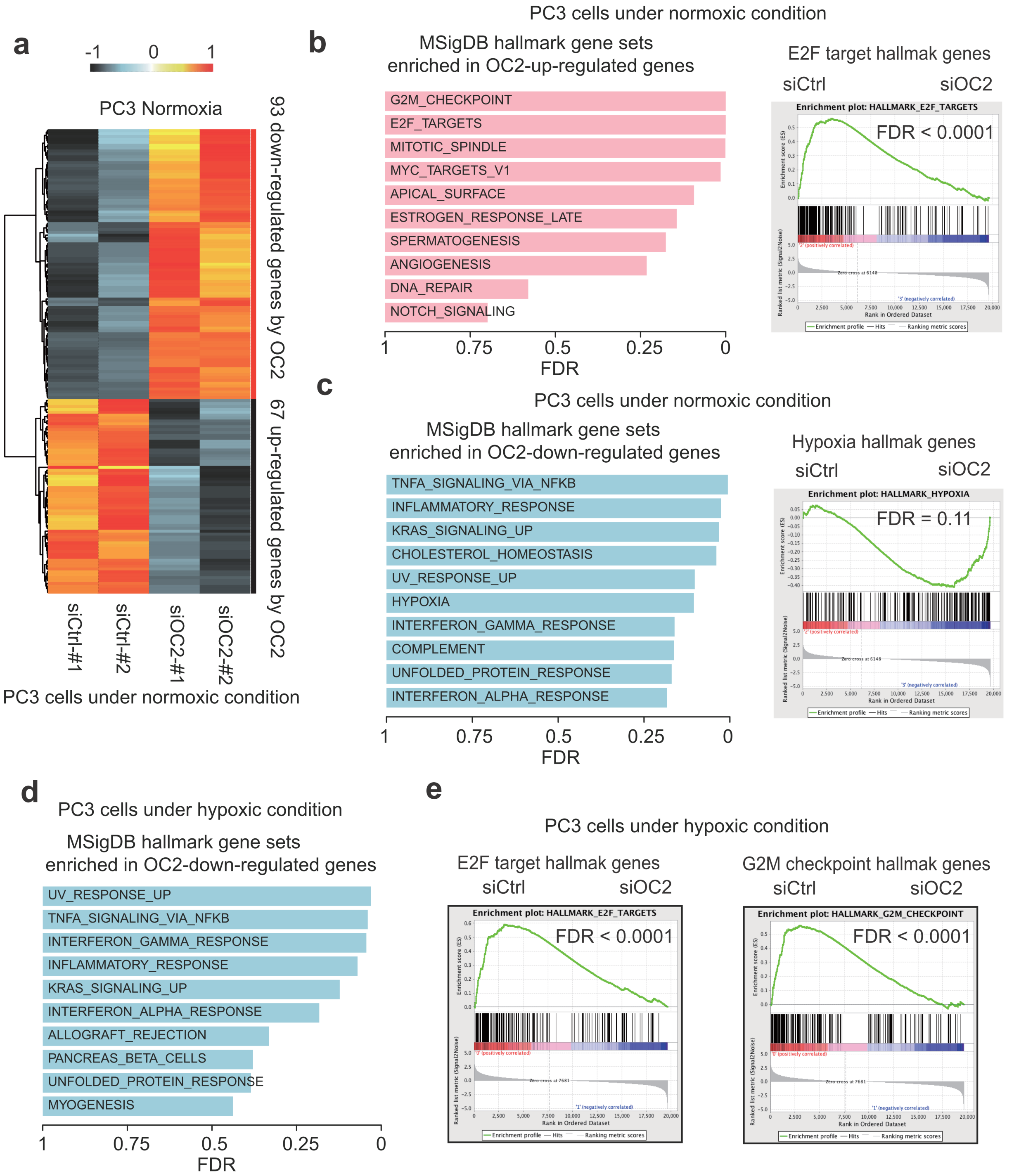

Supplementary Figure 5. ONECUT2 target genes in PC3 cells under normoxic and hypoxic conditions. (a) Heatmap shows expression of ONECUT2 target genes (absolute fold change > 2 and P < 0.05) in PC3 cells under normoxic conditions. (b) Left panel: Top 10 MSigDB Hallmark Gene Sets enriched in ONECUT2 up-regulated genes in PC3 cells under normoxic conditions. Right panel: Selected GSEA enrichment plot for "E2F targets" gene set. Genes were ranked by fold changes between control and ONECUT2 knockdown samples in descending order. (c) Left panel: Top 10 MSigDB Hallmark Gene Sets enriched in ONECUT2 down-regulated genes in PC3 cells under normoxic conditions. Right panel: Selected GSEA enrichment plot for

“Hypoxia” gene set. Genes were ranked by fold changes between control and ONECUT2 knockdown samples in descending order. (d) Top 10 MSigDB Hallmark Gene Sets enriched in ONECUT2 down-regulated genes in PC3 cells under hypoxic conditions. (e) Selected enrichment plots for “E2F targets” and “G2M checkpoint” gene sets enriched in ONECUT2 up-regulated genes under hypoxic conditions.

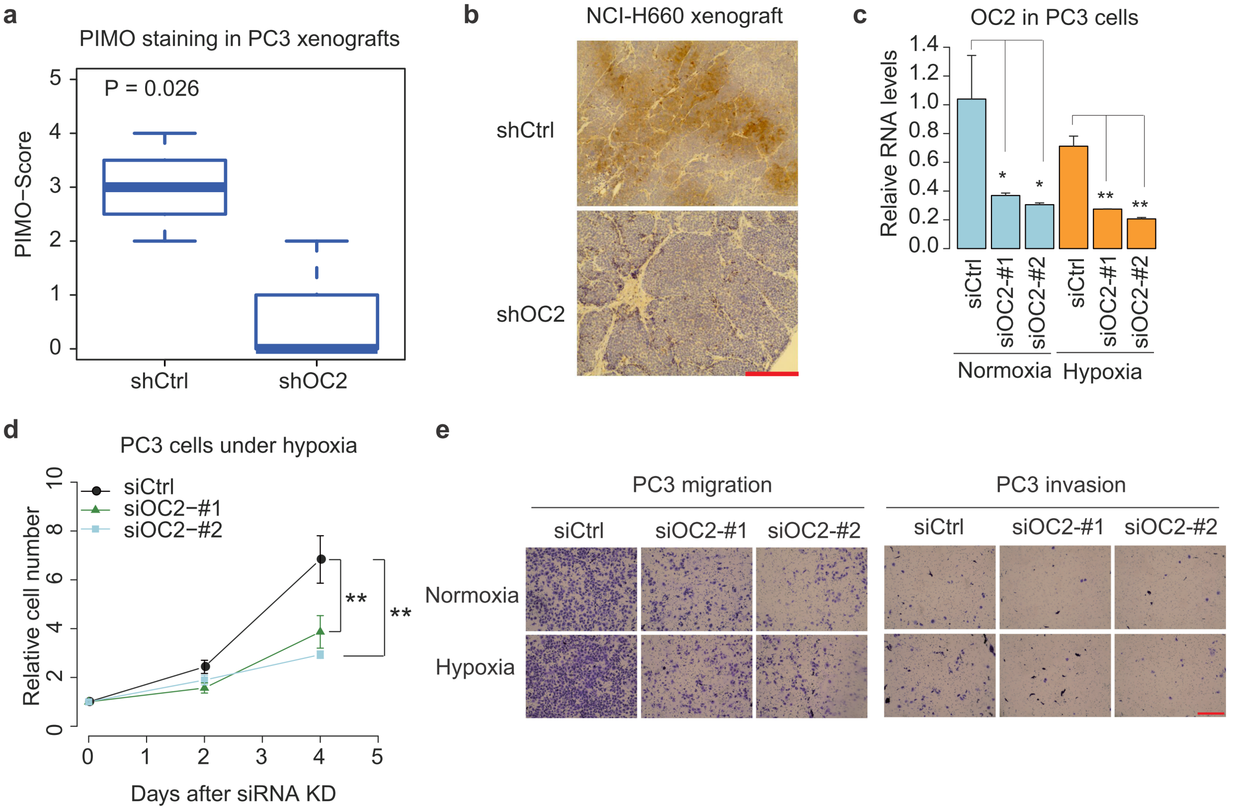

Supplementary Figure 6. ONECUT2 regulates hypoxia-induced aggressive phenotypes of PCa cells. (a) PIMO IHC of PC3 xenografts with and without silencing of ONECUT2. (b) Representative images of PIMO IHC staining of NCI-H660 xenograft tumors with and without ONECUT2 knockdown. Scale bar = 200 µm. (c) ONECUT2 mRNA levels after ONECUT2 knockdown under normoxic and hypoxic conditions. (d) PC3 cell proliferation in response to ONECUT2 siRNA knockdown under hypoxic conditions. (e) Representative images of PC3 cell migration and invasion. Scale bar = 100 µm. P-values were calculated from one-way ANOVA; **: P< 0.01. Source data are provided as a Source Data file.

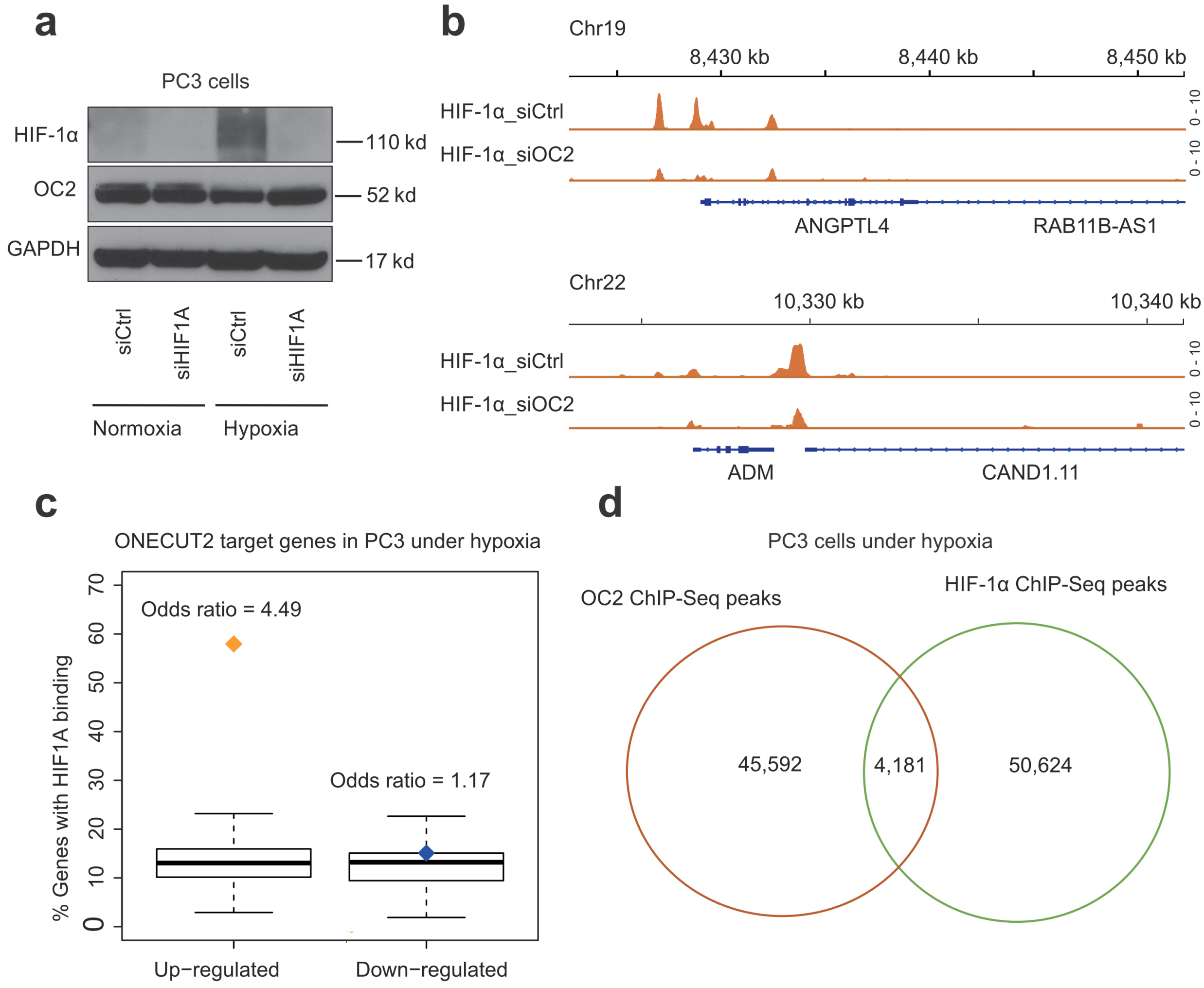

Supplementary Figure 7. HIF1α occupancy at hypoxia-induced genes in PC3 cells is regulated by ONECUT2. (a) Western blot of HIF1α and ONECUT2 in PC3 cells under hypoxic conditions. Two different siRNAs targeting the same gene were mixed together for knockdown experiments. (b) HIF1α ChIP-Seq signal at the promoter regions of hypoxia-induced genes ANGPTL4 and ADM. (c) Percentage of ONECUT2 regulated genes with HIF1α ChIP-Seq peaks nearby (± 20 kb) the transcription starting sites under hypoxic conditions. Orange and blue points represent ONECUT2 up-regulated and down-regulated gene sets, respectively. Boxes represent 1,000 times random sampling from the whole transcriptome. Same number of genes as in the up-regulated and down-regulated gene sets were used for random sampling, respectively. Odds ratio was calculated by comparing the percentage between ONECUT2 regulated gene sets and the average of random sampling. (d) The overlap of ONECUT2 ChIP-Seq and HIF1α ChIP-Seq peaks in PC3 cells under hypoxia conditions. Source data are provided as a Source Data file.

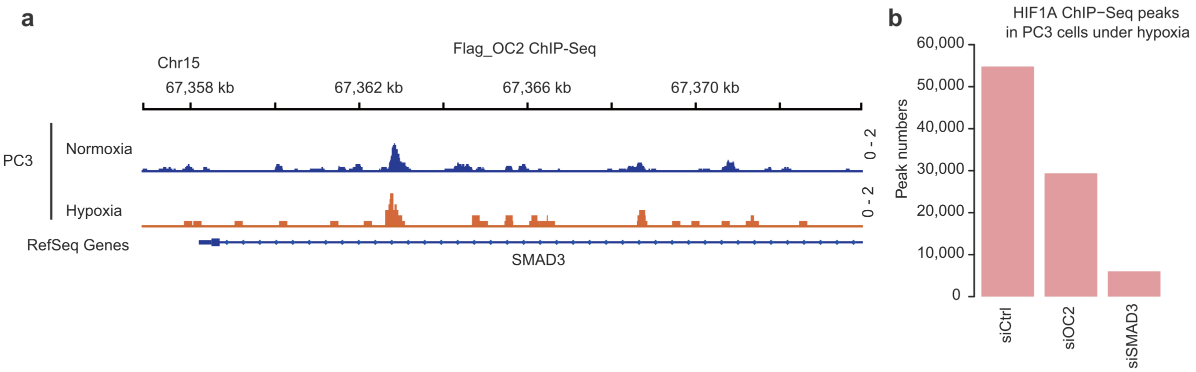

Supplementary Figure 8. SMAD3 regulates HIF1α recruitment to chromatin. (a) ONECUT2 binding sites at SMAD3 promoter region. (b) HIF1α binding sites were decreased upon silencing of ONECUT2 or SMAD3.

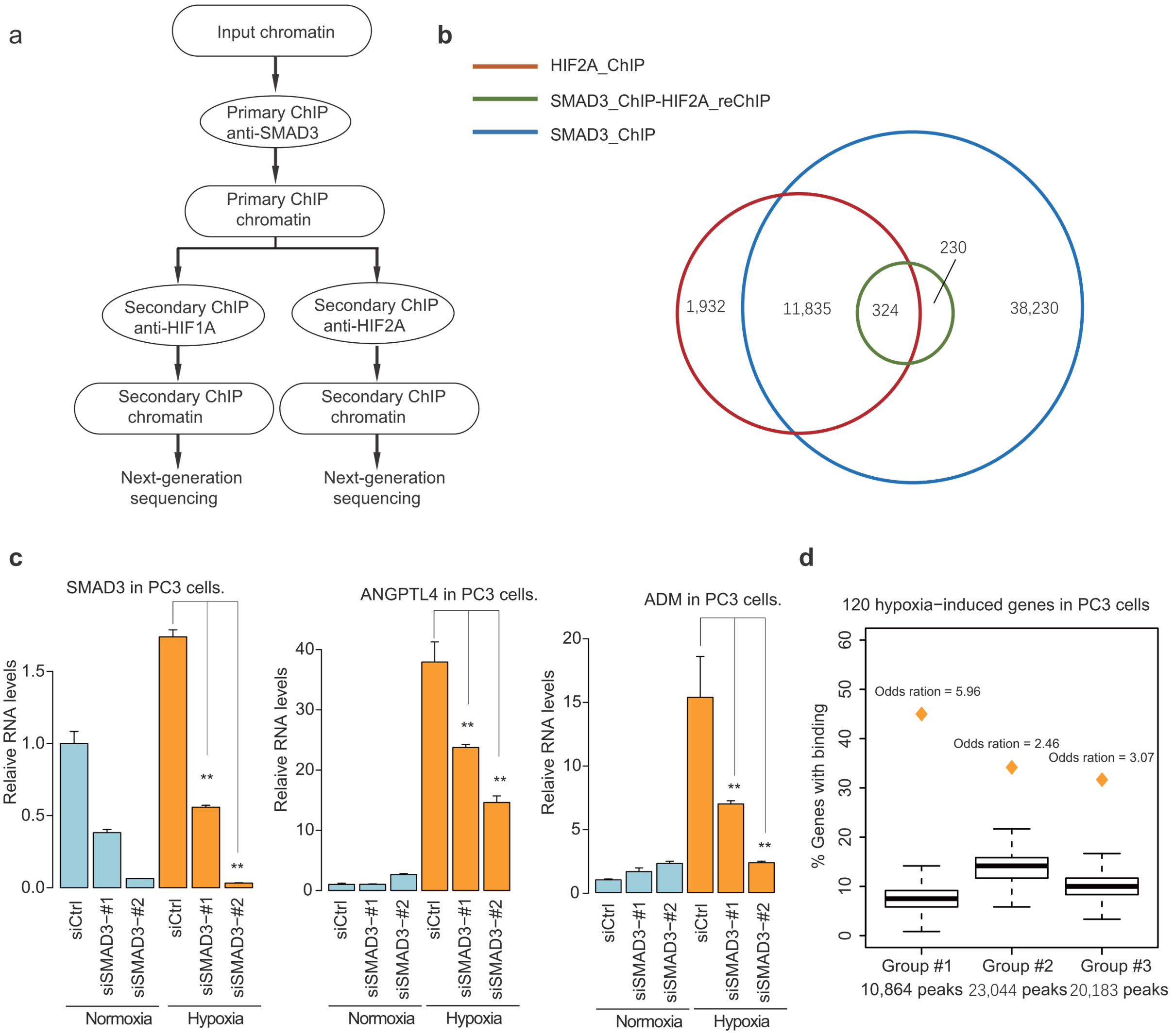

Supplementary Figure 9. SMAD3-HIF1α and SMAD3-HIF2α ChIP-re-ChIP sequencing. (a) The workflow of SMAD3 primary ChIP followed by HIF1α and HIF2α reChIP-Seq. (b) Overlapping of SMAD3-HIF2α reChIP-Seq peaks, SMAD3 ChIP-Seq peaks and HIF2α ChIP-Seq peaks. (c) SMAD3 is required for hypoxia induction of ANGPTL4 and ADM. P values were calculated by one-way ANOVA; **: $P < 0.01$. (d) HIF1α reChIP-Seq peaks overlapped with both HIF1α and SMAD3 ChIP-Seq peaks (Group1, Figure 3g) are more enriched in hypoxia-induced genes than other HIF1α peaks (Group2 and Group3, Figure 3g). Source data are provided as a Source Data file.

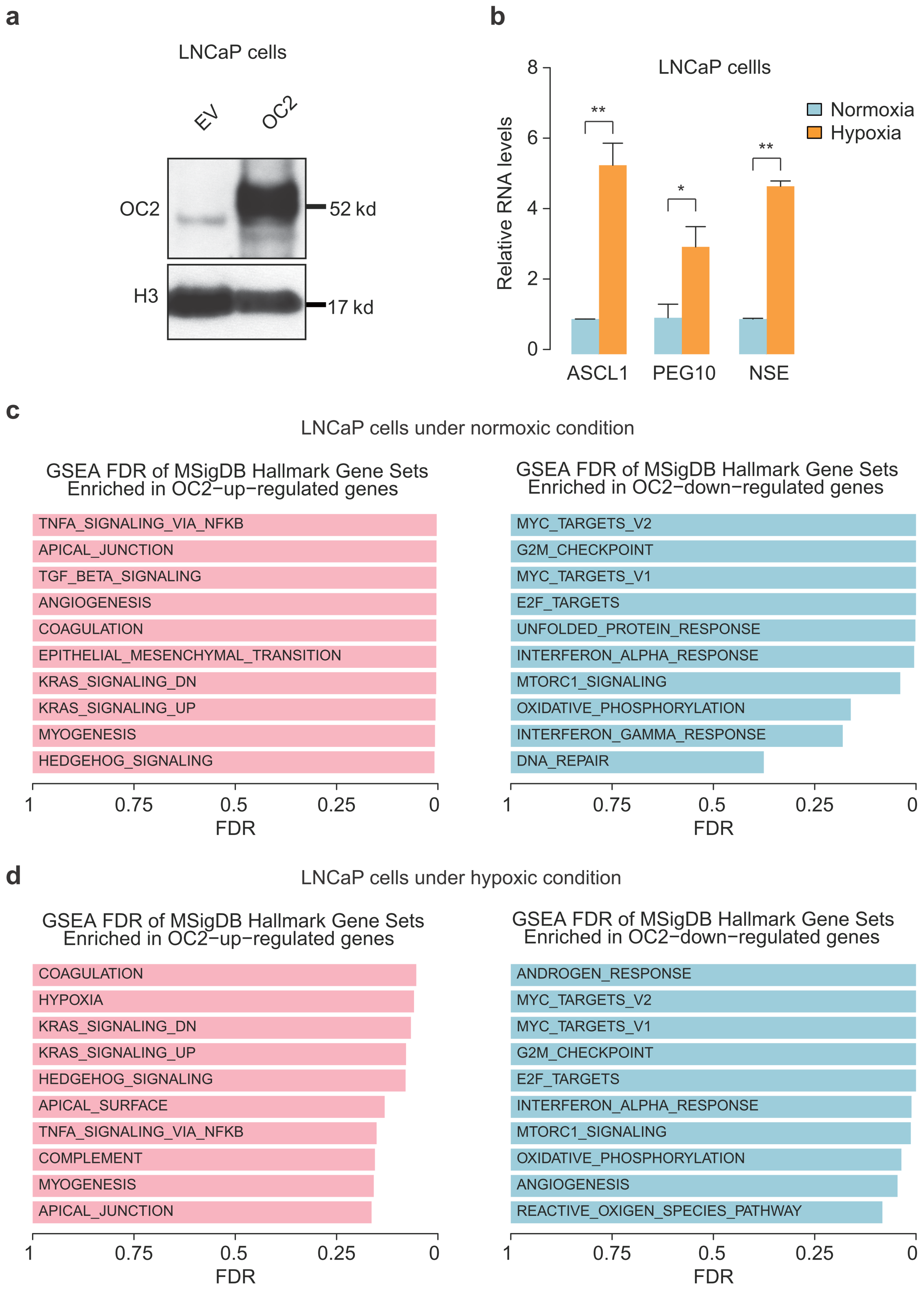
a
LNCaP cells
EV
OC2
OC2
H3
52 kd
17 kd
b
LNCaP cellls
Normoxia
Hypoxia
Relative RNA levels
0
2
4
6
8
**
*
**
ASCL1
PEG10
NSE
c
LNCaP cells under normoxic condition
GSEA FDR of MSigDB Hallmark Gene Sets
Enriched in OC2−up−regulated genes
TNFA_SIGNALING_VIA_NFKB
APICAL_JUNCTION
TGF_BETA_SIGNALING
ANGIOGENESIS
COAGULATION
EPITHELIAL_MESENCHYMAL_TRANSITION
KRAS_SIGNALING_DN
KRAS_SIGNALING_UP
MYOGENESIS
HEDGEHOG_SIGNALING
1
0.75
0.5
0.25
0
FDR
GSEA FDR of MSigDB Hallmark Gene Sets
Enriched in OC2−down−regulated genes
MYC_TARGETS_V2
G2M_CHECKPOINT
MYC_TARGETS_V1
E2F_TARGETS
UNFOLDED_PROTEIN_RESPONSE
INTERFERON_ALPHA_RESPONSE
MTORC1_SIGNALING
OXIDATIVE_PHOSPHORYLATION
INTERFERON_GAMMA_RESPONSE
DNA_REPAIR
1
0.75
0.5
0.25
0
FDR
d
LNCaP cells under hypoxic condition
GSEA FDR of MSigDB Hallmark Gene Sets
Enriched in OC2−up−regulated genes
COAGULATION
HYPOXIA
KRAS_SIGNALING_DN
KRAS_SIGNALING_UP
HEDGEHOG_SIGNALING
APICAL_SURFACE
TNFA_SIGNALING_VIA_NFKB
COMPLEMENT
MYOGENESIS
APICAL_JUNCTION
1
0.75
0.5
0.25
0
FDR
GSEA FDR of MSigDB Hallmark Gene Sets
Enriched in OC2−down−regulated genes
ANDROGEN_RESPONSE
MYC_TARGETS_V2
MYC_TARGETS_V1
G2M_CHECKPOINT
E2F_TARGETS
INTERFERON_ALPHA_RESPONSE
MTORC1_SIGNALING
OXIDATIVE_PHOSPHORYLATION
ANGIOGENESIS
REACTIVE_OXIGEN_SPECIES_PATHWAY
1
0.75
0.5
0.25
0
FDR

Supplementary Figure 10. ONECUT2 synergizes with HIF1α in driving neuroendocrine plasticity in prostatic adenocarcinoma. (a) ONECUT2 western blotting with and without overexpression of ONECUT2 in LNCaP cells. EV: empty vector control; OC2: ONECUT2 overexpression. (b) mRNA levels of NEPC marker genes in LNCaP cells under normoxic and hypoxic conditions. (c) Top 10 MSigDB Hallmark Gene Sets enriched in ONECUT2 up-regulated (pink bars) or down-regulated (blue bars) genes in LNCaP cells under normoxic conditions. (d) Top 10 MSigDB Hallmark Gene Sets enriched in ONECUT2 up-regulated (pink bars) or down-regulated (blue bars) genes in LNCaP cells under hypoxic conditions. Source data are provided as a Source Data file.

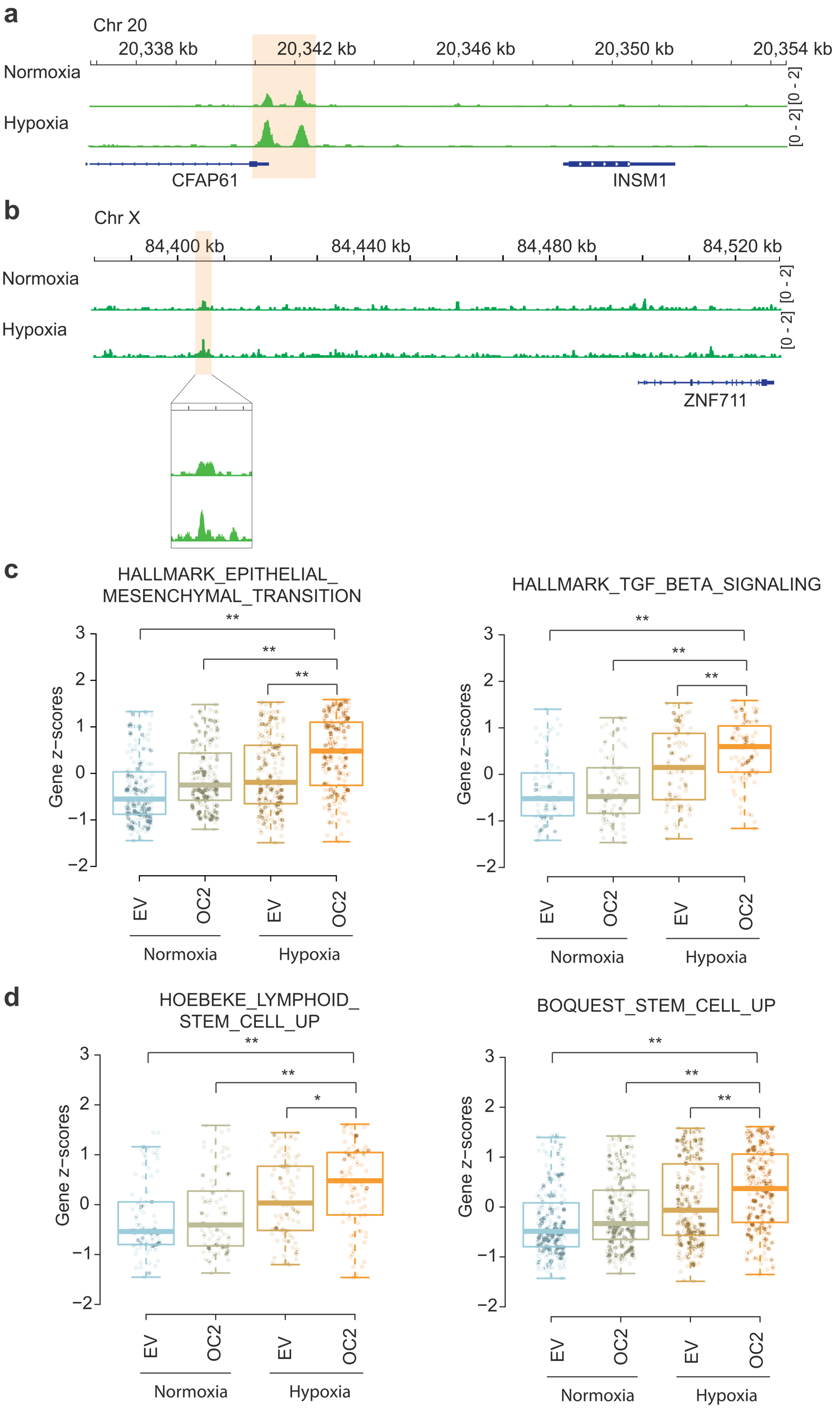
a
Chr 20
20,338 kb
20,342 kb
20,346 kb
20,350 kb
20,354 kb
Normoxia
Hypoxia
[0 - 2]
[0 - 2]
CFAP61
INSM1
b
Chr X
84,400 kb
84,440 kb
84,480 kb
84,520 kb
Normoxia
Hypoxia
[0 - 2]
[0 - 2]
ZNF711
c
HALLMARK_EPITHELIAL_
MESENCHYMAL_TRANSITION
HALLMARK_TGF_BETA_SIGNALING
Gene z−scores
EV
OC2
Normoxia
Hypoxia
d
HOEBEKE_LYMPHOID_
STEM_CELL_UP
BOQUEST_STEM_CELL_UP
Gene z−scores
EV
OC2
Normoxia
Hypoxia

Supplementary Figure 11. ONECUT2 synergizes with hypoxia to induce lineage plasticity in LNCaP cells. (a-b) Green tracks: ChIP-Seq signal of ONECUT2 in LNCaP cells under normoxic and hypoxic conditions. ONECUT2 Peaks surrounding INSM1 (a) and ZNF711 (b) are highlighted with orange boxes. (c) Z-scores of the two epithelial-mesenchymal transition (EMT)-related gene signatures from MSigDB calculated in LNCaP RNA-Seq data. RNA-Seq experiments were performed in LNCaP cells with and without ONECUT2 overexpression under normoxic and hypoxic conditions. (d) Z-scores of the two stem cell-related gene signatures from MSigDB calculated in LNCaP RNA-Seq data. P-values are calculated from Wilcoxon rank sum test. *: $P< 0.05$. **: $P< 0.01$. EV: empty vector control; OC2: ONECUT2 overexpression.

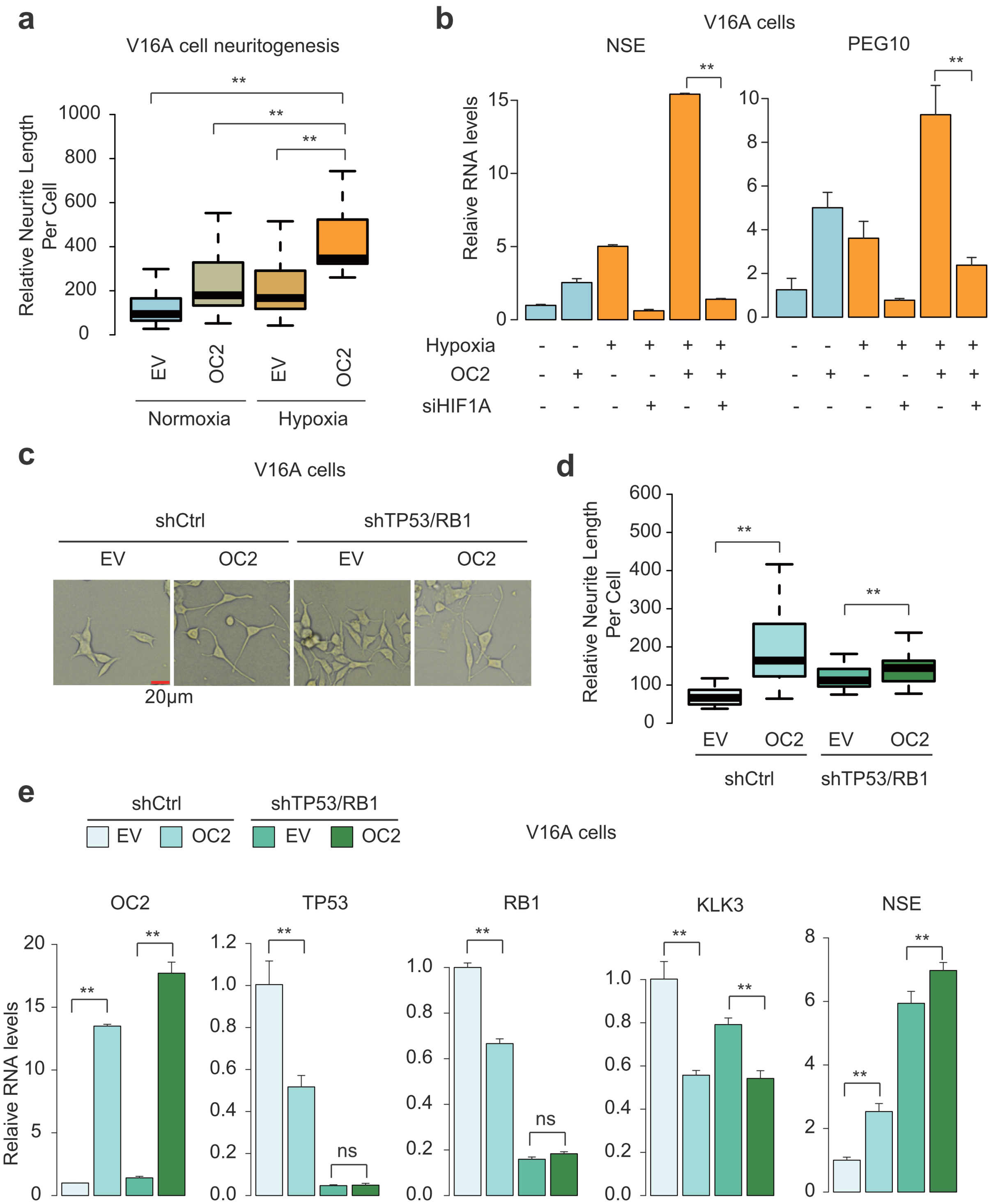

Supplementary Figure 12. ONECUT2 synergizes with hypoxia to induce neuroendocrine plasticity in V16A cells. (a) Neuritogenesis analysis in V16A cell with and without overexpression of ONECUT2 under normoxic and hypoxic conditions. P-values are calculated from Wilcoxon rank sum test. **: P< 0.01. (b) NSE and PEG10 mRNA expression in V16A cells. (c-d) Neuritogenesis analysis in V16A cell with and without overexpression of ONECUT2 in the background of TP53/RB1 double knockdown. P-values are calculated from Wilcoxon rank sum

test. **: P< 0.01. (e) Gene expression in V16A cells determined by RT-qPCR. For (b) and (e), error bars indicate s.d. from three technical replicates. P values were calculated by one-way ANOVA; **: P < 0.01. Source data are provided as a Source Data file.

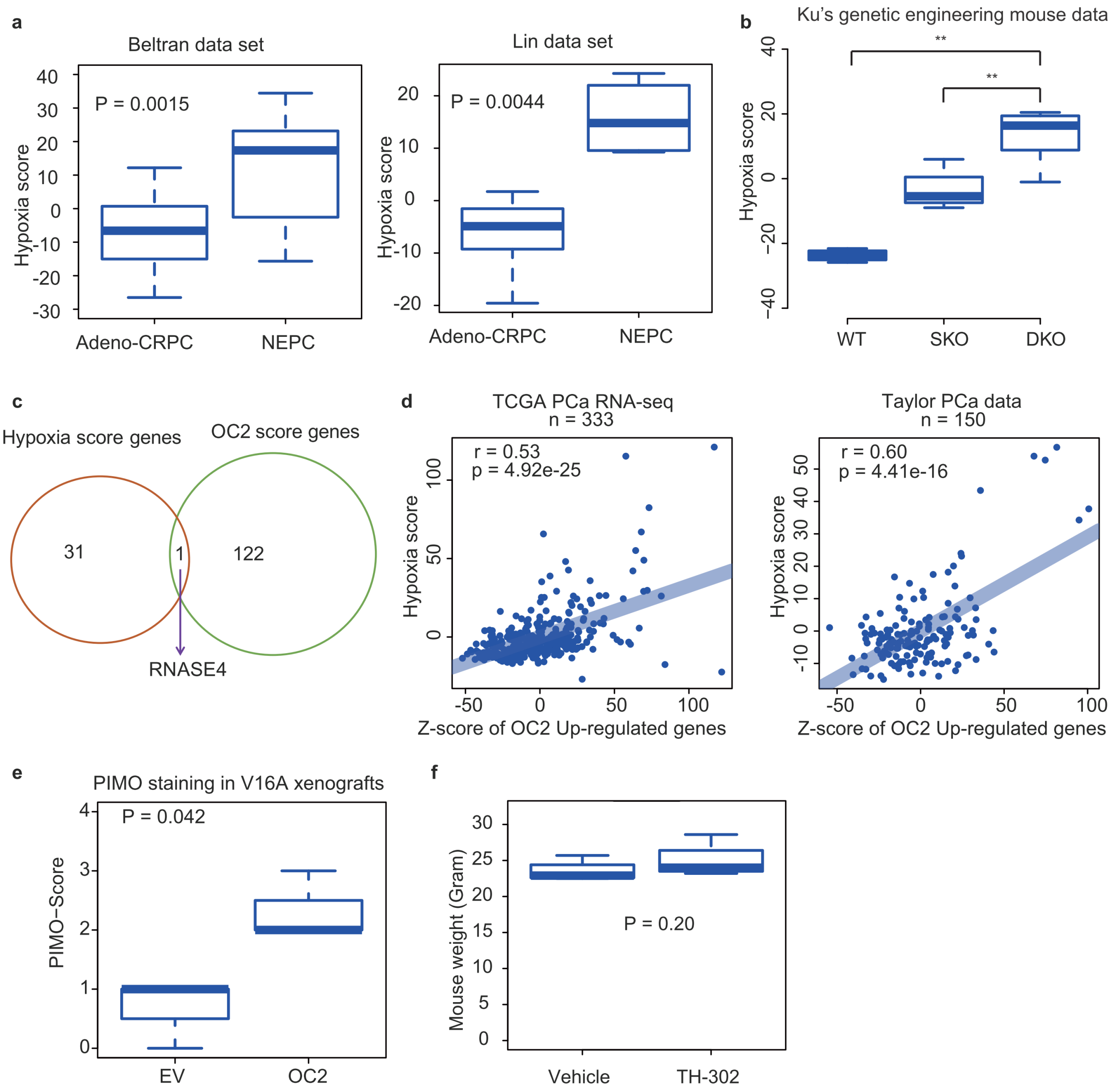

Supplementary Figure 13. ONECUT2 target genes are correlated with tumor hypoxia. (a) Hypoxia signature gene z-scores are higher in NEPC compared with adeno-CRPC in Beltran and Lin data sets. (b) Hypoxia signature gene z-scores are higher in DKO PCa tumors compared with SKO PCa tumors and WT prostate tissues in genetic engineered mouse models. For (a) and (b), P-value is determined by Wilcoxon rank sum test. (c) Venn diagram shows there is only one overlapping gene between the 32 Hypoxia signature genes and ONECUT2 up-regulated genes under hypoxic conditions. (d) Scatter plots show the correlation between ONECUT2-up-

regulated genes z-scores and hypoxia z-scores in TCGA and Taylor PCa data sets. (e) PIMO IHC of V16A xenograft tumors with and without overexpression of ONECUT2. (f) Body weight of vehicle and TH-302 treated NEPC PDX bearing mice. For (e-f), P-values were calculated based on Student's t-test.

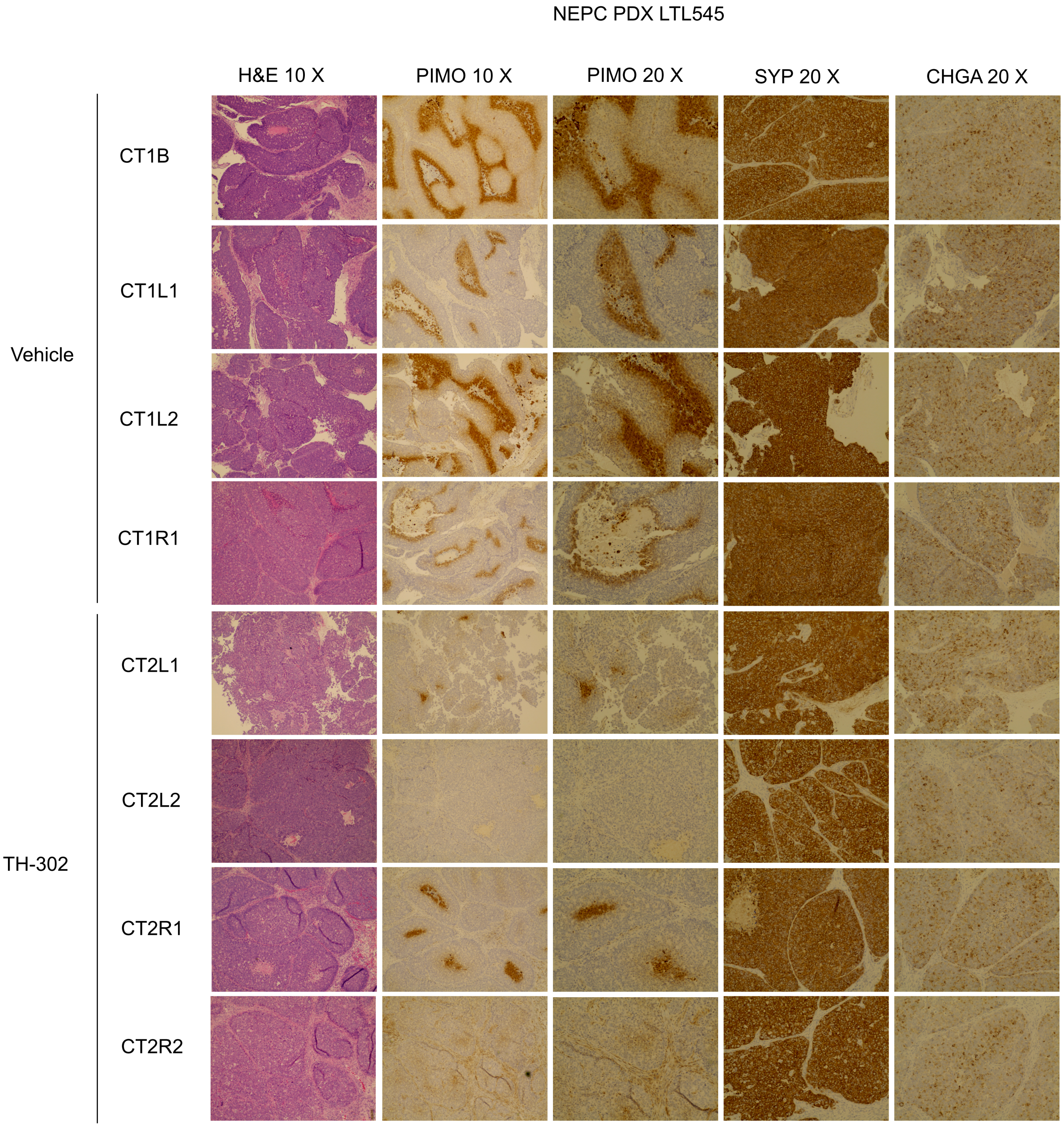

Supplementary Figure 14. Targeting hypoxia in NEPC. NEPC PDX model LTL545 was re-implanted in 8 mice, and the mice were treated with saline control or TH-302 when PDX tumor volume reached ~ 50 mm^3. After two weeks of treatment, PDX tumors were collected and subject to hematoxylin and eosin (H&E), PIMO, SYP and CHGA staining.

Supplementary table 1: 88 pan-NET genes in four data sets.

	Log2 fold change (SCLC vs. NSCLC)	Log2 fold change (neuroblastoma vs. glioma)	Log2 fold change (NEPC vs. adeno-CRPC)_Beltran	Log2 fold change (NEPC vs. adeno-CRPC)_Lin
ADAM22	1.23	1.29	1.43	3.10
ADCYAP1	1.92	2.28	2.81	8.23
AP3B2	1.66	1.45	2.28	5.10
ASCL1	2.94	3.17	5.21	6.61
ASXL3	2.00	1.19	2.33	6.88
ATP1A3	1.13	1.90	3.47	4.26
BEND5	1.96	1.42	1.43	3.14
BSN	1.62	1.81	1.52	3.29
CA10	1.64	1.22	1.59	6.98
CCDC33	1.23	1.38	2.30	2.02
CDK5R2	1.76	2.07	3.27	1.13
CELF4	1.82	2.33	1.90	5.75
CHGA	2.41	3.64	4.13	9.94
CHGB	1.83	2.24	3.06	7.80
CHRNB2	2.38	2.49	1.26	8.39
CPLX2	1.47	3.15	3.32	2.78
CRTAC1	1.59	1.58	1.51	4.74
CRYBA2	1.58	1.11	2.99	4.60
CXXC4	1.47	1.93	2.30	6.20
DCX	2.23	2.64	2.08	6.92
DDX25	2.04	2.00	1.55	2.80
DLGAP3	1.82	1.82	1.75	4.01
DLL3	1.70	2.24	5.68	7.26
DSCAML1	1.26	1.01	2.35	7.32
ELAVL4	2.58	3.36	2.74	6.98
FAM184A	1.17	1.34	1.45	2.20
FGF14	2.35	1.43	1.31	6.61
FXYD6	2.39	2.40	1.54	2.24
GNAO1	1.68	1.29	2.83	4.72
GNG4	1.29	1.90	2.05	3.23
IGFBPL1	1.60	2.68	2.30	3.54
INA	1.23	2.66	3.03	3.91
INSM1	2.99	2.25	4.21	8.05
KCNC1	2.01	2.24	1.53	5.31
KCNH2	1.88	2.48	1.89	2.56
KCNH6	2.18	1.27	2.98	5.37

KIAA1211	1.34	1.63	1.22	1.92
MARK1	1.07	1.35	1.27	2.67
MAST1	1.13	1.60	2.73	3.88
MTMR7	1.65	1.36	1.49	3.68
MTSS1	1.15	1.29	1.29	1.87
MYT1	2.42	2.57	1.97	4.30
MYT1L	2.47	2.51	1.08	4.52
NCAM1	2.17	1.24	2.71	2.94
NELL1	2.40	1.80	3.05	2.60
NRSN1	2.43	3.09	2.15	3.10
NRXN1	2.52	1.46	2.20	4.67
OGDHL	1.24	1.78	2.86	3.75
ONECUT2	1.37	1.63	2.59	4.57
PCSK2	2.88	1.04	3.06	8.26
PDZD4	1.61	1.20	1.61	2.43
PGBD5	1.46	1.05	1.85	5.33
PHYHIPL	2.04	3.11	1.32	4.64
PROX1	2.30	1.87	3.14	6.53
PTPRN	1.36	1.19	2.07	2.18
RAB3C	2.02	2.86	1.39	3.80
RAPGEF5	1.13	1.80	1.18	1.92
RGS16	1.63	1.57	1.64	4.72
RIMBP2	2.15	3.01	2.90	4.14
RIMKLA	1.24	1.26	2.07	1.74
RIMS2	1.37	1.35	1.31	4.44
RUNDC3A	2.08	2.65	1.75	4.30
RUNX1T1	2.46	1.31	1.29	5.21
SCGN	2.53	1.55	3.50	6.96
SCN3A	2.16	1.17	3.56	1.68
SEZ6	2.83	2.13	3.10	4.31
SHD	1.71	3.57	1.40	1.93
SIX2	1.24	1.43	3.09	3.86
SLC35D3	1.80	1.26	3.13	9.31
SNCB	1.66	1.16	1.63	1.40
SPTBN4	1.38	1.28	2.94	2.28
SRRM3	1.13	1.26	1.78	2.90
SRRM4	2.03	2.13	2.14	9.07
ST8SIA3	2.03	2.26	1.67	2.46
STXBP5L	2.14	1.41	1.14	2.91
SVOP	2.20	2.38	1.17	6.43
SYN1	2.14	1.89	1.79	3.21
SYP	2.00	2.10	2.56	4.63
SYT4	2.81	2.95	2.40	8.00

TAGLN3	2.43	1.39	3.69	3.12
TMEM151B	1.54	2.19	1.92	2.53
TMEM63C	1.04	1.17	1.97	5.85
TMOD2	1.25	1.04	1.29	2.21
UNC13A	1.87	2.27	2.58	5.78
UNC5A	1.55	2.09	1.77	3.55
VGF	1.02	2.06	4.02	7.31
XKR7	2.47	2.38	2.07	3.05
ZNF711	1.36	1.52	2.04	5.14

Supplementary table 2: 154 motifs enriched in LNCaP HIF1A ChIP−Seq of OC2 unique peaks vs. EV unique peaks

id	consensus	E.value	adj_p.value
AHR	DTYGCGTGM	2.90E-36	3.70E-39
AP2B	GCCYGVGGGS	4.30E-03	5.60E-06
AP2D	CGCCYGVGGCSCGT	4.20E+00	5.40E-03
ARNT2	GNCGTGGSASRC	2.20E-08	2.90E-11
ARNT	STACGTGMC	1.90E-169	2.40E-172
ATF1	HRRTGACGYMA	3.50E-55	4.60E-58
ATF2	RRTGABGTCAY	1.70E-06	2.20E-09
ATF3	GGTSACGTGAB	1.70E-46	2.20E-49
ATF6A	GKGSTGACGTGG	2.50E-10	3.30E-13
ATF7	KRTGACGTCAT	1.10E-04	1.50E-07
BACH1	TGCTGAGTCABSS	8.90E-77	1.10E-79
BACH2	TGCTGAGTCAB	2.10E-132	2.70E-135
BATF	RBTYTCRDWATGASTCAB	4.40E-16	5.80E-19
BHE40	DGCACGTGAS	1.60E-04	2.10E-07
BMAL1	KSCACGTGHSY	1.30E-04	1.70E-07
CLOCK	CWGHCACGTGMVVM	1.90E-05	2.50E-08
CR3L1	GCCACGTGGCA	3.50E-05	4.50E-08
CR3L2	TGATGACGTGGCA	1.20E-61	1.60E-64
CREB1	NRRTGACGTMA	2.20E-54	2.80E-57
CREB3	KGRTGACGTGGCA	9.40E-16	1.20E-18
CREB5	RRTGACGTMAT	1.60E-08	2.10E-11
CREM	CRVTGACGTCA	4.00E-02	5.20E-05
CTCFL	GCCDSYAGGGGGCGCYV	6.80E-05	8.80E-08
CTCF	YGGCCACCAGRKGGCRSYV	2.80E-02	3.60E-05
E2F2	GGCGCGAAAC	4.50E-05	5.80E-08

E2F3	DDGGCGGGAAA	7.10E+00	9.20E-03
E2F4	SRGGGCGGGAARD	6.80E-14	8.90E-17
E2F7	GDGGCGGGAARDR	1.80E-01	2.30E-04
E4F1	YGTKACGTC	2.30E-27	3.00E-30
EGR1	VDRDGCGKGGGYGGRRR	1.70E-04	2.20E-07
EGR2	GRGRRKGWGKGGGHGGRG	1.30E-01	1.60E-04
EGR4	GGSGGYRGGGM	1.50E-04	1.90E-07
EHF	VWACSAGGAAGTDVS	1.20E-20	1.60E-23
ELF1	VRACCCGGAAGTGS	5.10E-31	6.60E-34
ELF2	RAVCCGGAAGTGR	1.80E-31	2.40E-34
ELF3	RAABVAGGAAGTRR	3.10E-08	4.00E-11
ELF5	VGRASSAGGAAGTRV	2.10E-10	2.80E-13
ELK1	RCCGGAAGTGV	3.00E-38	3.80E-41
ELK3	VMCHGGAARTSC	3.50E-18	4.50E-21
ELK4	SVCCGGAAGTGV	9.30E-44	1.20E-46
EPAS1	VTACGTGMC	1.40E-161	1.90E-164
ERG	RRRSAGGAAGYGG	4.30E-21	5.60E-24
ESR1	AGGTCASMSTGACCY	2.50E+00	3.30E-03
ESR2	RGGTCASCSTGMCCY	2.00E+00	2.60E-03
ETS1	RRRCMGGAAGTGG	2.50E-32	3.20E-35
ETV1	SRCCGGAAGYG	1.60E-42	2.00E-45
ETV2	RRARRCAGGAARYRGS	1.10E-20	1.40E-23
ETV4	VRCAGGAARBR	4.20E-07	5.40E-10
ETV5	RRRSAGGAARDGRV	7.50E+00	9.70E-03
ETV6	RCAGGAARK	1.40E-06	1.80E-09
ETV7	VAGGAAR	4.30E-03	5.60E-06
FEV	GCVGGAAGYG	3.70E-17	4.70E-20
FLI1	SRRGGMAGGAAGGRRRGR	5.50E+00	7.20E-03
FOSB	RTGAGTCAB	1.90E-242	2.40E-245
FOSL1	KRVTGAGTCAYH	2.50E-302	3.20E-305
FOSL2	NDRTGAGTCAYH	2.40e-314	3.10e-317
FOS	VTGAGTCAB	1.00E-255	1.30E-258
GABPA	GVVRCCGGAAGTGV	6.50E-36	8.40E-39
GLI3	BTGGGTGGTCY	5.50E+00	7.10E-03
GLIS1	TCGTGGGGGGTMD	2.10E+00	2.70E-03
GMEB2	NBKTACGTVRN	7.00E-40	9.10E-43
HES1	VMGVCACGMGMCM	9.20E-05	1.20E-07
HES5	YGGCACGTGYCR	5.70E-03	7.40E-06
HES7	TGGCACGTGCCG	3.50E-01	4.50E-04
HESX1	ABSYGGCWMRTRRMMT	1.20E-05	1.50E-08
HEY1	KGRCACGTGBCK	3.30E-35	4.20E-38
HIC2	HCRTGCCAGVN	2.10E-02	2.80E-05
HIF1A	GDACGTGM	5.30E-127	6.90E-130

HINFP	DMSHHMGCGGACGTTV	5.00E+00	6.50E-03
INSM1	TGTMAGGGGGCR	6.40E+00	8.30E-03
JDP2	BGATGACGTCAY	1.70E-10	2.20E-13
JUNB	DVTGAGTCABH	1.10E-255	1.50E-258
JUND	RRTGAGTCAYY	1.00E-273	1.30E-276
JUN	DRTGAGTCAYH	5.90E-276	7.70E-279
KAISO	YTSGCRGGARKH	2.30E-01	3.00E-04
KLF12	VGGGGCGGGGC	1.80E-26	2.40E-29
KLF13	DRGKGGGCGKD	2.50E-07	3.30E-10
KLF14	DRGKGGGCGTGGY	2.10E-17	2.70E-20
KLF15	RGGGMGGRGVDGGGGGRGG	4.10E-02	5.30E-05
KLF16	GGGGGYGKGKBGGGGGGGG	3.00E-07	3.90E-10
KLF1	DGGGYGKGGCYGGG	1.40E-30	1.80E-33
KLF3	GGRGVDGGGCGGGGCYGGG	1.20E-26	1.60E-29
KLF4	WGGGYGKGGC	2.80E-32	3.70E-35
KLF5	WGGGTGKGGCDGGG	9.10E-22	1.20E-24
KLF6	GSRRGGGHGGGGMHGGGRV	1.80E-10	2.30E-13
KLF8	CAGGGKGTG	5.70E-04	7.40E-07
KLF9	GRRGKGGGCGTGGCC	1.00E-23	1.30E-26
MAFB	TGCTGASTYAD	3.20E-18	4.10E-21
MAFF	TGCTGASTCAGCABTTTT	2.10E-09	2.70E-12
MAFG	RWWWNTGCTGASTCAGCA	4.30E-20	5.60E-23
MAFK	RAWWHTGCTGASTCAGCA	7.10E-38	9.10E-41
MAF	VAAWBTGCTGASTHWGCMD	5.60E+00	7.30E-03
MAX	VRGCACGTGG	1.70E-04	2.20E-07
MAZ	RRGGGAGGGGS	6.00E-07	7.80E-10
MBD2	SSGKCCGGMGR	7.70E-03	1.00E-05
MECP2	SCCGGRR	1.40E+00	1.80E-03
MEIS3	DKTGACAGSTK	3.30E+00	4.30E-03
MITF	TCACGTGACC	3.20E-04	4.20E-07
MXI1	VCACGTGGSNGSBGS	5.10E-06	6.70E-09
MYCN	RGCCACGTGSDS	1.20E-08	1.50E-11
MYC	RCCACGTGCTB	2.10E-07	2.70E-10
NF2L1	HGTCATN	2.70E-61	3.50E-64
NF2L2	WWYTGCTGAGTCAT	6.80E-156	8.80E-159
NFE2	VVRTGACTCAGCA	3.20E-145	4.20E-148
NFIA	TTGGCHNRNWGCCAR	1.70E-06	2.20E-09
NFIB	YCYTGGCAS	7.40E-05	9.60E-08
NFIC	CYTGGCHBYMWGCCARG	6.60E-05	8.60E-08
NR1H4	RRGGKCAVTGRCCBSGVGG	7.10E-03	9.20E-06
NRF1	CWSTGCGCATGCGCRDS	4.10E-05	5.40E-08
NRL	TGCTGACKYWR	2.80E-23	3.60E-26
P63	VDRCATGCCTG	3.10E-03	4.00E-06

PAX2	RHTCAGTSAYGMGTGAYW	2.60E-29	3.40E-32
PAX5	SCSKGGGCARCVRAGCGWGAC	4.80E-02	6.20E-05
PAX8	BTVAYTSRMGYRKR	1.20E-07	1.60E-10
PBX1	TGABTGACAG	8.80E-03	1.10E-05
PBX3	TGABTGRCRGS	2.40E-14	3.20E-17
PDX1	TGATTGATK	3.90E-13	5.10E-16
PEBB	TYTGTGGTYWB	1.20E+00	1.60E-03
PKNX1	TGABTGRCAGS	7.80E-06	1.00E-08
PROX1	KGGKARGGCGKSKKGGG	7.50E-01	9.80E-04
RORG	RRAASTRGGTCA	1.80E-46	2.30E-49
RUNX1	BYTGTGGTTWB	1.10E-07	1.40E-10
RUNX2	KGKYTGTGGTTTKK	1.90E-02	2.40E-05
RUNX3	BYTGTGGTTW	1.40E-08	1.80E-11
SALL4	GSDGGGWGGG	1.80E-04	2.40E-07
SMAD3/4	CWGTCTGDCACCT	5.70E-01	7.40E-04
SP1	VGGGGGCGGGGCCKGGGGGGG	7.70E-22	1.00E-24
SP2	GGSSVGGGGGCGGGGCCDGSGS	1.10E-08	1.50E-11
SP3	SGVVGGGGGCGGGGCBRGSS	2.30E-16	3.00E-19
SP4	SRGVARGRGGCGGRGCHDRR	1.30E-04	1.70E-07
SPDEF	RWCCMGGAWGWAGT	3.40E-03	4.40E-06
SPI1	RRAAAGAGGAAGTGRVD	3.10E+00	4.10E-03
SPIB	RRAAAGAGGAAGTGARA	7.30E-04	9.50E-07
SRBP1	DGTSRGGTGAB	5.00E-10	6.50E-13
SRBP2	VSGTGGGGWGABG	2.40E-05	3.10E-08
TBX15	GGGGGGGGGGGGTGGGDR	1.90E-01	2.50E-04
TFCP2	SCCTGMNCDSRCCRGA	1.60E+00	2.10E-03
TFDP1	RRRRGGCGGGAARN	2.20E+00	2.90E-03
TFE3	RGTCACGTGA	3.60E-13	4.70E-16
TFEB	RGTCACGTG	8.80E-29	1.10E-31
THAP1	SGCCGCCATSTYGGSYBCGGGC	8.90E-02	1.20E-04
USF2	GGGTCACGTGRBSVSSSSB	3.20E-02	4.10E-05
WT1	RGGVGGGGGAGGRGGVGGRG	1.80E+00	2.30E-03
XBP1	GSKGACGTGGC	4.40E-30	5.70E-33
ZBT14	GGAGCGCGC	3.90E-05	5.00E-08
ZIC3	GGCYHCCTGCTGWGH	5.80E-03	7.50E-06
ZN148	KGRGKGGGGGAGGGG	8.00E-06	1.00E-08
ZN219	GDGGGGGGYGGA	1.20E-05	1.50E-08
ZN281	RGGWGGGGGAGGGGV	2.10E-01	2.70E-04
ZN335	YGCCTGW	5.00E-03	6.50E-06
ZN554	GCWGAGYCANGTRGDKKRCT	1.90E-26	2.50E-29
ZN563	SHRGCAGCWSWG	1.20E-02	1.60E-05
ZN740	DKKGGGGGGGHRDK	2.70E+00	3.50E-03
ZSC31	RGCAGCAGGGCARTTAKR	6.00E-01	7.80E-04

Supplementary table 3: Sequences of primers, siRNAs and shRNAs.

qPCR primers	
ADM-F	CTTGGTGACACTGGATAGAACA
ADM-R	CACGACTCAGAGCCCACTTA
ANGPTL4-F	CCTGGGACGAGATGAATGT
ANGPTL4-R	TGAGCCTTGAGTTGTGTCTG
OC2-F	AGAGTCTGCCCAACTACGGT
OC2-R	CGTTCAGGTGCGACATCAT
TP53-F	CATTCTGGGACAGCCAAGT
TP53-R	TTTCCTTCCACTCGGATAAG
RB1-F	TTCCTCCACACACTCCAGT
RB1-R	CGGTAATACAAGCGAACTCC
KLK3-F	TGTGTGCTGGACGCTGGA
KLK3-R	CACTGCCCCATGACGTGAT
NSE-F	CCGGGAACTCAGACCTCATC
NSE-R	CTCTGCACCTAGTCGCATGG
ASCL1-F	CCCAAGCAAGTCAAGCGACA
ASCL1-R	AAGCCGCTGAAGTTGAGCC
PEG10-F	ATCCTTCCTGTCTTCGCA
PEG10-R	ACACACGCACTCTTATGGC
SMAD3-F	TGACCACCAGATGAACCAC
SMAD3-R	ACATTGGAGAGCAGCCCTA
U6-F	CTCGCTTCGGCAGCACA
U6-R	AACGCTTCACGAATTTGCGT
siRNA	
siONECUT2-#1	CAAACGCAAAGAGCAAGAAtt
siONECUT2-#2	GCAAAGAGCAAGAACCAAAtt
siHIF1A-#1	CCAUAUAGAGAUACUCAAAtt
siHIF1A-#2	CCUCAGUGUGGGUAUAAGAtt
siSMAD3-#1	GCCUGGUCAAGAAACUCAAtt
siSMAD3-#2	AGGUCUGCGUGAAUCCCUAtt
shRNA	
shONECUT2-#1	GCCATGAACAACCTCTACAGT
shONECUT2-#2	CAACCTCTACAGTCCCTACAA
