# Supplementary material for: ONECUT2 is a driver of neuroendocrine prostate cancer
Source: Nat Commun. 2019 Jan 17;10:278. doi: 10.1038/s41467-018-08133-6 (PMC6336817; doi:10.1038/s41467-018-08133-6)
Supplement: Supplementary file 2 — Description of Additional Supplementary Files [file 41467_2018_8133_MOESM2_ESM.docx]

**Description of Additional Supplementary Files**

**File Name**: Supplementary Data 1

**Description**: Source data for Figs 2g, 2h, 3a, 3c, 3e, 4b, 5e, 5g and 5h and Supplementary Figs 4a, 4b, 4c, 4f, 6c, 7a, 9c, 10a, 11b and 11e.
